# Relating macrofungal diversity and forest characteristics in boreal forests in China: Conservation effects, inter‐forest‐type variations, and association decoupling

**DOI:** 10.1002/ece3.8049

**Published:** 2021-08-27

**Authors:** Wenjie Wang, Jingxue Sun, Zhaoliang Zhong, Lu Xiao, Yuanyuan Wang, Huimei Wang

**Affiliations:** ^1^ Urban Forests and Wetlands group Northeast Institute of Geography and Agroecology Chinese Academy of Science Changchun China; ^2^ Key Laboratory of Forest Plant Ecology Ministry of Education Northeast Forestry University Harbin China

**Keywords:** association decoupling, larch forests, macrofungi taxonomy and functional guilds, National Nature Reserves, plant compositional and structural traits

## Abstract

**Question:**

How conservation and forest type affect macrofungal compositional diversity is not well understood. Even less is known about macrofungal associations with plants, soils, and geoclimatic conditions.

**Location:**

Southern edge of boreal forest distribution in China, named as Huzhong Nature Reserve.

**Methods:**

We surveyed a total of 72 plots for recording macrofungi, plants, and topography in 2015 and measured soil organic carbon, nitrogen, and bulk density. Effects of conservation and forest types on macrofungi and plants were compared, and their associations were decoupled by structural equation modeling (SEM) and redundancy ordination (RDA).

**Results:**

Conservation and forest type largely shaped macrofungal diversity. Most of the macrofungal traits declined with the conservation intensities or peaked at the middle conservation region. Similarly, 91% of macrofungal traits declined or peaked in the middle succession stage of birch‐larch forests. Forest conservation resulted in the observation of sparse, larch‐dominant, larger tree forests. Moreover, the soil outside the Reserve had more water, higher fertility, and lower bulk density, showing miscellaneous wood forest preference. There is a complex association between conservation site characteristics, soils, plants, and macrofungi. Variation partitioning showed that soil N was the top‐one factor explaining the macrofungal variations (10%). As shown in SEM coefficients, conservation effect to macrofungi (1.1–1.2, *p* < .05) was like those from soils (1.2–1.6, *p* < .05), but much larger than the effect from plants (0.01–0.14, *p* > .10). For all tested macrofungal traits, 89%–97% of their variations were from soils, and 5%–21% were from conservation measures, while plants compensated 1%–10% of these effects. Our survey found a total of 207 macrofungal species, and 65 of them are new updates in this Reserve, indicating data shortage for the macrofungi list here.

**Conclusion:**

Our findings provide new data for the joint conservation of macrofungi and plant communities, highlighting the crucial importance of soil matrix for macrofungal conservation in boreal forests.

## Graphical abstract

SEM analysis of the conservation effects on macrofungi (diversity and functional groups) and possible indirect pathways from soil and plants. Study site location was at Huzhong Reserve at NE China, the southernmost edge boreal forests of the world.

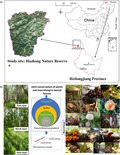

**Highlights**

1. Conservation declined or middle‐peaked 78% of tested macrofungal traits.
2. Succession from secondary to climax larch forests declines 43% of tested macrofungal traits.
3. Conservation induced sparse, pure, and larger tree forests, and moist, fertile, and light soil was outside the Reserve.
4. 89%–97% of macrofungal variations were from soils and N was the top‐one factor, and 5%–21% were from conservation, while plants compensated 1%–10% of these effects.
5. Macrofungal conservation in boreal forests should pay more attentions to soils matrix selection.

## 1 INTRODUCTION

Macrofungi are fungi with fruiting bodies observable by the naked eye. They have been recognized as ecosystem resources for conservation owing to their fundamental positions in ecosystems functioning, including nutrient cycles and wood decomposition, or exploitation for human utilization (edible food and pharmaceutical medicine ingredients) (Wu et al., 2019; Zotti et al., 2013). Forest is the primary habitat for various macrofungi. Plant species have been used as indicators or surrogates for microfungal conservation (Mcmullan‐fisher et al., 2010) and assessment of macrofungal species richness (Schmit et al., 2005). Macrofungal diversity is responsible for various ecosystem functions of soil carbon cycling, plant nutrition, pathology, and plant tolerance to abiotic and biotic stress (Tedersoo et al., 2020). The macrofungal compositional difference in natural forests has been studied in taxonomic species, functional guilds at the regional (Zhang et al., 2010), and national level across China (Wu et al., 2019). These variations were dependent on forest types and habitats (Mölder et al., 2020), topographical environment (Caiafa et al., 2017; Kutszegi et al., 2015), and altitude and growth matrix (Mcmullan‐fisher et al., 2010; Ranius et al., 2019; Zhang et al., 2010). Although this advances, compared with plants and animals, we still have many unknown taxonomic composition of macrofungi (at least 35,000 species, with known lists about 21,679) (Mueller et al., 2007). How to conserve this huge macrofungal biodiversity get more and more concerns (Li et al., 2021; May et al., 2019; Minter et al., 2012; Senn‐Irlet et al., 2007).

Daxinganling Mts. is the only boreal forest region in China, and the total area is 0.323 Million km^2^ at the cold temperate region at the border between China and Russia, and most of the region is covered by larch climax natural forests (Wang et al., 2021; Zhou, 1997). Different studies have been done in this region, identifying the importance of this region for the ecological shelter of NE China, such as carbon sequestration (Xiao et al., 2020), wildfire recovery (Xu et al., 1997), plant diversity conservation (Yang et al., 2019; Zhang et al., 2018), and soil nutrient dynamics (Chen et al., 2019). Forest succession in this region was generally characterized as secondary miscellaneous wood forests, birch‐larch forests, and larch climax forests. The conservation intensity in Nature Reserve in China can be described as the strongest intensity at core region (strict human‐disturbance prohibition), buffer region (lower intensity than the core region), experimental region (scientific experiment can be done here only), and outside the Reserve (no protection at all) (Wang et al., 2021; Zhong, 2021). Daxinganling Mts. also supports about 2.0 million people (80% in Inner Mongolia and 20% in Heilongjiang Province) for their life. Natural forest Protection Program (NFPP) has recognized this region as the top priority region without timber harvest since 2000 (Zhang et al., 2000), and wild macrofungus collection has become a vital economic resource for local households in this region (Zhu et al., 2017). However, the macrofungal study in this region still focused on the list of macrofungus species for utilization (Bau & Li, 2010; Bau et al., 2019; Deng, 2010; Deng & Sun, 2010; Deng & Wang, 2010; Shao & Xiang, 2017; Wu et al., 2019; Xiang, 2005; Yu et al., 2005), wood‐decaying function (Cui et al., 2019), and wildfire‐fungal successions (Yang et al., 2020), with minimal concerns on their conservation. Together with the multiple analyses, full utilization of various taxonomic species (Bau et al., 2019), functional guilds (habitat and utilization) (Du et al., 2017; Heilmann‐Clausen & Christensen, 2005; Wu et al., 2019), and various diversity indices (Du et al., 2017; Ruiz‐Almenara et al., 2019), could favor a holistic view of macrofungal characteristics and prefer a conservation‐utilization sustainable way in the boreal forest region.

Huzhong Nature Reserve located in the central region of Daxinganling Mts. This Reserve is one of the oldest natural reserves in China (Zhuang, 2010). Under this background, a detailed investigation of the vegetation characteristics and macrofungi could favor the define of the conservation effect on macrofungi and the complex association with plants and conservation measures (different conservating intensities from the completely human‐disturbance prohibition in the Core Reserve region to nonconservation outside the Reserve).

Our hypothesis is that conservation and forest succession strongly affect macrofungal diversity, and the effects were strongly shaped by conservation measures, soil matrix, and plants owing to their complex associations. We want to explore the following questions:

1. How much difference between different conservation intensity (outside the Reserve, buffer, experimental, and core Reserve region) and different forest types (secondary miscellaneous wood forests, birch‐larch forests, and larch climax forests)?
2. What are the differences in geographical location, soil properties, and plant traits among conservation intensity and forest types? What is their association with macrofungal characteristics?
3. Possible implications for macrofungi conservation in NE China?

The fulfillment of the current knowledge gap of macrofungi–plant–conservation–soil associations will favor the conservation of forest macrofungi and sustainable field collection to support local economic development. Our data will provide a new basis for local macrofungi resource utilization and prefer the precise evaluation of natural forests during NFPP implementation in China.

## 2 MATERIALS AND METHODS

### 2.1 Study site and experimental design

**FIGURE 1:**
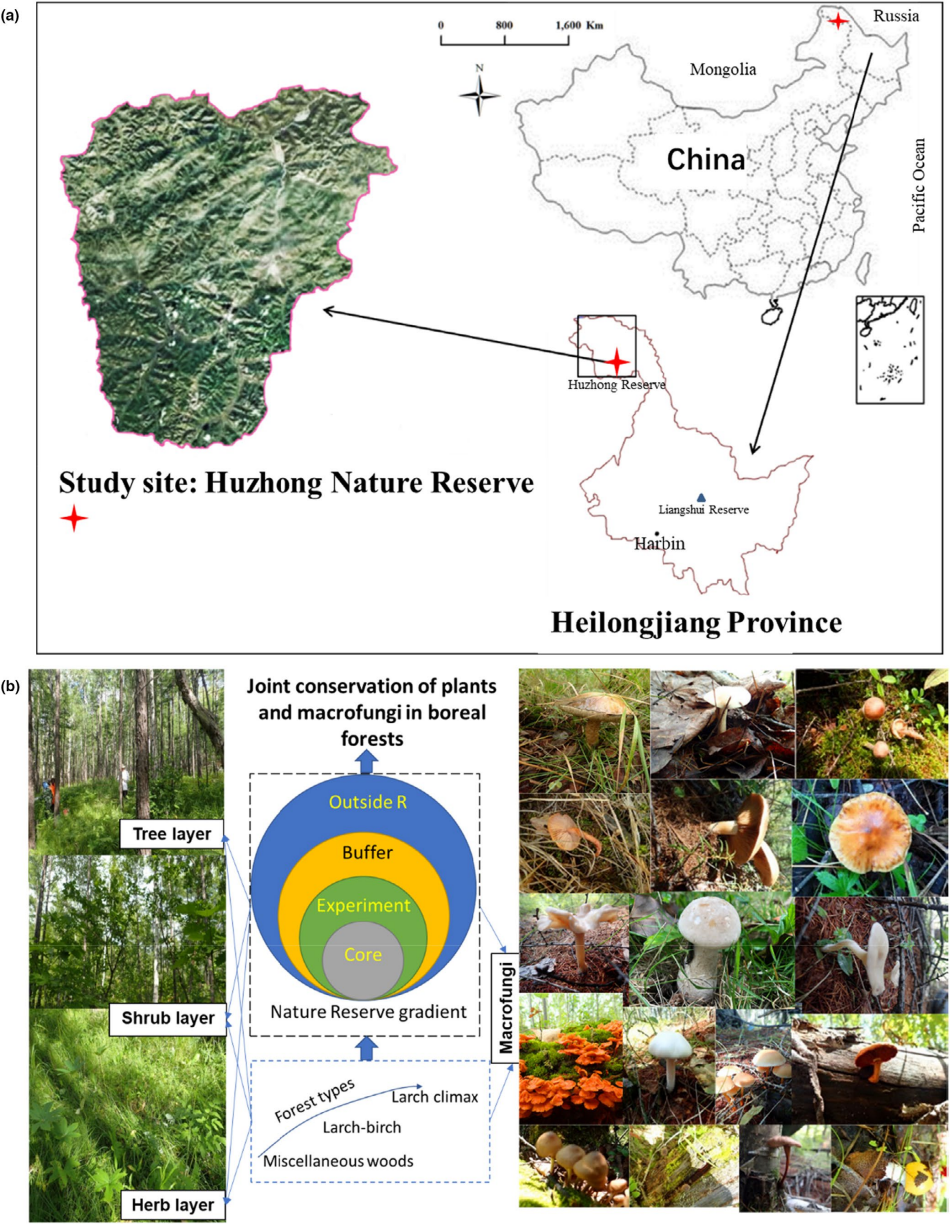
Location of the Huzhong Nature Reserve, relative to China map and Heilongjiang Province map. Harbin, the capital city of Heilongjiang Province, and another Reserve (Liangshui) were also shown on the Heilongjiang province map (a), and the basic experimental design of this experiment (b), that is, comparison on the forest type and conservation gradients on plants and macrofungi, and also decoupling their associations

The study sites were in the Huzhong National Natural Reserve and adjacent region, located in the Daxinganling Mts. region (122°83'86″E–123°70'43″E; 51°63'62″N–52°29'67″N; 469–982 m.a.s.l.) (Figure 1). This Reserve is in the northernmost province of China (Heilongjiang Province), and neighbors to Russia, and the total area of this Reserve is 167,213 ha (Figure 1). The prominent topography is the basic tectonic structure dominated by low hills, surrounded by mountains and gullies, with complex mountainous terrain. The climate is a cold‐temperate continental monsoon climate, with annual mean precipitation of 497.7 mm and an annual mean temperature of −4.3°C. Forest coverage here is nearly 90%, and total timber volume is 3.97 × 10^7^ m^3^ with climax forest of larch forests (Zhou, 1997). In the last 50‐year human timber harvest, deciduous pioneer birch trees have become more and more dominant nowadays (Wang et al., 2021).

Experimental design for identifying conservation effects and forest‐type effects is as the following (Figure 1). According to conservation intensities from low to high, 25 plots were located outside the Reserve, 16 plots were in the buffer region inside the Reserve, and 13 plots were in the experimental area inside the Reserve, while 18 plots were in the core region of the Reserve. The plots were also classified according to forest types from the most disturbing class in this region (miscellaneous wood forests with various broadleaved trees and grasses), birch‐larch forests, to the climax forests (larch forests). In miscellaneous wood forests, a total of 28 plots was surveyed; in birch‐larch forests, a total of 14 plots was surveyed, and in larch climax forests, a total of 30 plots was surveyed. In sum, a total of 72 plots (30 m × 30 m) was surveyed in August–September 2015.

Among the four conservation intensities, the digital number was scored to the core area, buffer area, experimental area, and outside the Reserve as 4, 3, 2, 1. According to the distance to human disturbance and the close to climax vegetation (succession stage), three forest types of miscellaneous wood forests, birch‐larch forests, and larch climax forests were scored as 1, 2, and 3, respectively. These scores made the redundancy ordination analysis (RDA) and structural modeling (SEM) between conservation measures and macrofungal traits available later.

### 2.2 Macrofungal survey for the taxonomic and functional guild and diversity index calculation

The species name, the total number of each macrofungus in each plot, and growing habitats (soil, litter, living tree, deadwood) were recorded in detail by at least three times cross‐line checking in the plots (30 m × 30 m in size). Macrofungi were identified by the morphological features with the help of microscope observation. In the morphological observation, naked eyes or magnifying lenses were used to check the color, shape, ancilla features of the hypophyll, pileus, mediotrastum, collyrium, stipe, volva, and rhizomorph on‐site, and also at least 5–8 digital photographs were taken for the later checks. For some fungi, spore prints from sporocarp were also collected, and Melzer's reagent was also used to tested amyloid (from blue to black) and dextrinoid (brown to red‐brown). All photographs were taken from different angles and specific interested anatomy. The images obtained were named according to the sample number, convenient for later laboratory recognition. The species identification in the field and the laboratory was carried out by referring to relevant literature and handbook (Chen et al., 2013; Huang, 1998; Liu, 2004; Mao, 2000; Nature‐museum‐editorial‐board, 2014; Shao & Xiang, 2017; Xiang, 2005; Yu et al., 2005). Reconfirming the identification was also achieved with the help of Prof. Cunti Xiang, a macrofungi expert in this region. The fungi were finally checked in the 10th edition of the fungus dictionary (Wirk et al., 2008), and the IndexFungorum online database (www.indexfungorum.org and www.speciesfungorum.org) was used for the classification, with final grouping all species into genus, family, and order for later analysis.

After the species recognition, all these macrofungi were divided into five utilization‐related functional guilds (edible macrofungi, medicinal macrofungi, toxic macrofungi, wood‐rot macrofungi, unknown‐function macrofungi) and five habitat‐related functional guilds (living wood macrofungi, deadwood macrofungi, soil‐based macrofungi, litter‐based macrofungi). Owing to the field‐collection purpose of local people, almost every textbook for macrofungi identification has included this functional guild classification. Thus, all the grouping was identified according to the textbooks for macrofungi in China (Chen et al., 2013; Huang, 1998; Liu, 2004; Mao, 2000; Shao & Xiang, 2017; Xiang, 2005; Yu et al., 2005). Given that some conflicts among different books, local textbooks were used as the classification criterion. These functional guilds were used to analyze the functional changes of macrofungi from habitat and utilization viewpoints.

We also calculated the species diversity index in this paper. The diversity of macrofungus was calculated by several methods commonly used in ecology (Bau & Li, 2000).

$$\text{Comprehensive}\;\text{index}\left(P_{i}\right)=\frac{1}{3}\left(A_{i}+B_{i}+C_{i}\right)$$

$$\text{Margalefrichnes}\;\text{index(MR)}\;\text{R =}\;\frac{S‐1}{\ln\;N}$$

$$\text{Shannon - - Wiener}\left(MH^{\prime }\right)\;H^{\prime }=‐\sum p_{i}\ln\;p_{i}$$

$$\text{Simpson}\;\text{index}\left(\text{MD}\right)D=1‐\sum p_{i}^{2}$$

$$\text{Pielou}\;\text{evenness}\;\text{index(ME)}E=‐\frac{\sum p_{i}\ln\;p_{i}}{\ln\;S}$$

where A_i_, B_i_, and C_i_ are the relative density (species *i* density/total density of the plot), relative abundance (species *i* number/total macrofungus number in the plot), and relative frequency (species *i* frequency in total surveyed plot/the number of plots). S and N are the total species number and individual number of macrofungus in the no. *i* plot.

### 2.3 Plot census for forest characteristics and conservation–soil matrix description

The plots were also used for plant census, soil sampling, and geographical–topographical determination during the macrofungus survey. For the tree layers, all trees >2.5 cm in the 30 m × 30 m plots were recorded by tree height, underbranch height, DBH (diameter at breast height), forest canopy density, and tree density. Tree species are also identified at the same time for plant species diversity calculation. For shrub layers, shrub species name, shrub density, shrub height, canopy width, and ground diameter were recorded in at least two subplots (2 m × 2 m) randomly settled in the 30 m × 30 m plot. For herb layers, five subplots (1 m × 1 m) were randomly settled in the 30 m × 30 m plot, and we recorded species name, herb coverage, species abundance, herb height, and herb density.

We also recorded the altitude, slope aspect (shade = 1, half‐sunny‐shade = 2, flat = 3, sunny = 4), slope position (valley = 1, low = 2, middle = 3, upper = 4 of the slope), and slope gradient in degree, latitude, and longitude of each sampling plot. The numbering of slope aspect and slope position could facilitate the RDA ordination of these parameters with macrofungal diversity variations.

The soil in the surface layer <20 cm after cleaning the recognizable litters was collected by a 100‐cm^3^ stainless‐soil‐cutting ring (4 cups/plot) and stored in a cloth bag ventilated until a constant mass. The differences between fresh and air‐dried soil were used to calculate soil moisture (differences/air‐dried soil, in percentage). Air‐dried soil mass in the cloth bag was used to calculate the soil bulk density (soil mass/volume = 400 cm^3^). The air‐dried soils were then grounded and used for laboratory determination of soil organic carbon and total nitrogen via the potassium dichromate volumetric method (external heating method) (Wang et al., 2018, 2019) and the semi‐micro‐Kjeldahl procedure (Wang, Lu, et al., 2017), respectively. A 20 cm × 20 cm frame‐cut and whole‐harvest measured litter amount at the forest floor into a paper bag for air‐dried measurement (at least three bags/each plot).

In this paper, forest characteristics were described by two types of parameters. The first type is forest structural attributes related to plant size and density. Parameters included tree height (th), underbranch height (ubh), diameter at breast height (DBH) at the tree layer; shrub height (sh), shrub coverage (sc), shrub canopy width (scw), shrub ground diameter (sgd) at the shrub layer, and the herb layer in herb height (hc), herb relative abundance (had), herb coverage (hc), and herb density (hd). $\text{Height}=\sum_{i=1}^{n}x_{i}/n$, and $\text{diameter}=\sum_{i=1}^{n}x_{i}/n$ (*n* is the total measured individual in the plot, xi is the height or diameter of the no. I individual). Canopy size is an average of canopy width, and canopy density is measured by the one‐step‐one‐head‐watching method (i.e., total canopy watching steps/the total steps with and without canopy watching). Density is the total number of plants in one unit of the ground surface (for tree and shrub, the total number were recorded in the field, while for herbs, an estimation of the total individual was approximately estimated and scaling‐up to one unit surface area). When different species were recorded, these parameters were averaged by different species with the weight of their relative abundance.

The second type of forest characteristic is plant species diversity. Plant diversity of richness index, diversity indices, and evenness indices was calculated with the field surveyed data (Ma et al., 1995).

$$\text{Patrickrichness}\;\text{index}\left(R\right):R=N$$

$$\text{Shannon - - Wiener}\;\text{index}\left(H^{\prime }\right)H^{\prime }=‐\sum p_{i}\ln\;p_{i}$$

$$\text{Pielouevenness}\;\text{index}\left(E\right)E=‐\frac{\sum p_{i}\ln\;p_{i}}{\ln\;S}$$

where P_i_ is the proportion of the number of species i to the total number of the forest, and *N* is the total number of species in the sampling plot. These diversity parameters were also, respectively, calculated as tree layer (TR, TH', TE), shrub layer (SR, SH', SE), and herb layer (HR, HH', HE).

### 2.4 Data analysis

For finding the variance in different conservation regions, the least significant difference method (LSD) and two‐way MANOVA were used to compare the significant differences of the total species number, plot‐average species richness, and species diversity of different functional guilds (edible, medicinal, toxic, wood‐rot, and unknown‐function macrofungi) and habitat guilds (living‐wood, deadwood, soil‐based, and litter‐habitat macrofungi) in different conservation intensity regions (core area, buffer area, experiment area, and outside the Reserve) and forest type (miscellaneous wood forest, birch‐larch forest, and larch forest).

For comparing the forest‐type differences and conservation effects, a new parameter was defined as the following: $\text{Effect}\;\text{size}=\left|\frac{\text{Average}(\text{Climax}+\text{Birch}‐\text{larch})}{\text{miscellaneous}\;\text{woods}}‐1\right|$ for different forest types or $=\left|\frac{\text{Inside}\;\text{Reserve}\left(\text{buffer},\;\text{Expe}.,\;\text{Core}\right)}{\text{Outside}\;\text{Reserve}}‐1\right|$ for different conservation intensities. The average of these effect sizes for different parameters was compared to find the relative size of conservation and forest types for macrofungi and other parameters. The larger effect sizes at statistical significance (*p* < .05) indicated that the parameters could strongly impact the treatments, or else no differences exist.

The macrofungal changing pattern at the conservation/succession gradients was identified by regression analysis. Four types of patterns were increasing, decreasing, middle‐peaked, and others. The best‐fitted regression of linear, log‐linear, exponential, power, and binomial equations was used when their *r* ^2^ is >.64 for conservation and >.85 for forest types. For linear, log‐linear, exponential, and power regression, the highest *r* ^2^ was selected for determining the changing pattern, and the negative coefficients indicated the decreasing pattern. In contrast, the positive one shows an increasing pattern. When binomial regression was selected and *r* ^2^ > .9, the changing pattern was middle‐peaked. Besides the regression analysis, we also used multiple comparison data for identifying the middle‐peaked pattern. In the middle of the conservation and the forest types, a significantly higher value in the medium conservation intensities or the middle stage of the succession indicated a middle‐peaked pattern, too.

Using Canonical redundancy analysis (RDA) to explore the coupling associations among macrofungi (different habitat, utilization function groups, taxonomy order, and family as well as diversity indices), forest characteristics, soil properties, and conservation measures (location selection, topography, forest types, and conservation intensities). The significant factors responsible for the macrofungal variation were statistically identified under the simple term effect and the conditional term effect (excluding colinear association among explaining factors). During the analysis, explaining factors were transformed by Log transformation and centralized during figure ordination by the software automatically.

SEM (structural equation model) analysis was used to find the pathway coefficient (direct effect and indirect effect) of the conservation measures (location, topography, forest type, and conservation intensity) on macrofungal traits and to find the possible association with plants and soils. A higher coefficient of the effect indicates a potentially more vital contribution to macrofungal conservation. For conservation measures, plants, and soils, a larger and more significant coefficient means a potentially more decisive contribution for conserving macrofungi. This criterion was used to select the most critical variables for macrofungal conservation in theory. Details of the SEM method can be found in previous publications (Wang, Zhong, et al., 2017; Wang et al., 2020; Zhong et al., 2017).

Analysis of variance and regression analysis was performed by the SPSS 22.0 (SPSS, USA). The RDA analysis was performed by the Canoco 5 (Biometrics, The Netherlands), and SEM was performed by the IBM SPSS AMOS 22.0 (SPSS, USA).

## 3 RESULTS

### 3.1 Conservation effects on the macrofungal functional guild, taxonomy group, and diversity

Of the total nine habitat and utilization functional guilds, 4 showed nonsignificant changes (*p* > .05) (Table A1 in Appendix S1). There were significantly higher edible macrofungi, medicinal macrofungi, and soil‐based macrofungi in the buffer or experimental region inside the Reserve (*p* < .05). In contrast, deadwood‐habitat and living wood‐habitat macrofungi declined with the increasing conservation intensities (Table A1 in Appendix S1). In general, the effect size ranged from 0.14‐fold to 0.48‐fold (Table A1 in Appendix S1).

For diversity indices, peak values of MH', MR, and ME were found at the experimental region inside the Reserve (Table A1 in Appendix S1). For MD, stronger conservating intensity is accompanied by a lower MD value (Table A1 in Appendix S1). From taxonomy order, a total of 8 orders were observed (Table A4 in Appendix S1), and conservation decreased the order number from 7 outside the Reserve to 4 inside the Reserve (buffer, experimental, and core regions). The most abundant orders were Agaricales and Polyporales. Species number in these two orders decreased 50%–60% inside the Reserve compared with those outside the Reserve. On average per one plot from these two orders, Agaricales peaked at the experimental region inside the Reserve, while Polyporales decreased with conservating intensities (Table A1 in Appendix S1). In the case of the taxonomy family, the higher conservation region accompanied the lower total family number. Inside the Reserve (experimental, buffer, and core region) was 2/3 of those outside the Reserve (21 families). On average of plot‐level data, the peak values were found in the buffer region in general (Table A1 in Appendix S1).

In all, conservation largely shaped macrofungal composition and diversity. In most cases, values peaked at medium‐protected region (9 parameters), or protection linearly decreased macrofungal traits (7 parameters). Linearly increased parameters were Boletaceae's and Cortinariaceae's number on average of plot‐level data. All other five parameters fluctuated with conservation or no significant changes at all (Table A1 in Appendix S1).

### 3.2 Forest type differences in macrofungi

In the case of utilization and habitat functional guilds, succession from miscellaneous woods to larch climax forests is accompanied by decreased medicinal, wood‐rot, toxic, unknown‐function macrofungi, and deadwood living wood‐ and litter‐habitat macrofungi. Some of them showed significant differences, such as medicinal macrofungi and deadwood‐habitat macrofungi (Table A2 in Appendix S1). Edible macrofungi and soil‐based macrofungi peaked at the birch‐larch forests. The difference (effect size) ranged from 0.11‐fold to 0.68‐fold (Table A2 in Appendix S1).

In the case of diversity indices, MR, MH', and ME middle‐peaked at the birch‐larch forests, while the succession of forest types induced declines in ME. All these differences were statistically significant in different forest types (*p* < .05), and effect sizes ranged from 0.08‐fold to 0.54‐fold (Table A2 in Appendix S1). The most dominant orders were Agaricales and Polyporales. There were 81–118 species from Agaricales in 3 forest types, and 21–24 species were from Polyporales (Table A2 in Appendix S1). In the family case, each forest had 17–19 families, and middle‐peaked values were in the birch‐larch woods, 2.2‐ to 2.5‐fold higher in plot data average than the other two forest types (Table A2 in Appendix S1).

In all, the forward succession of forest types accompanied with macrofungal declines in 11 parameters, and macrofungal middle‐peaked in the birch‐larch forests in another ten parameters. The increasing pattern was found in Boletaceae and Cortinariaceae's family number per one plot (Table A2 in Appendix S1).

### 3.3 Geo‐topography, soil properties, plant traits in forest types, and conservation regions

The more vigorous conservation intensity of the Reserve is usually located at high altitude regions in the southeast direction (lower latitude and longitude). However, no differences were found in the slope features (position, direction, and degree) (Table A3 in Appendix S1). The more southeastern region (lower longitude and latitude) distributed the larch climax forests (core region). In contrast, the west area with the lower altitude spread the more miscellaneous wood forests (Table A3 in Appendix S1).

For soil properties outside the Reserve usually had the moist, N‐fertile, high SOC, and porous soil (low bulk density), although the differences were generally not significant (*p* > .05). In different forest types, miscellaneous wood forests usually had more soil water, higher N and SOC, and lower bulk density (Table A3 in Appendix S1).

The conservation intensity from the low to the high (core region) did not induce tree height increases, but an evident rise in DBH and a sharp decline in tree density; during this process, shrub became short with increased coverage (but no significant changes in shrub density, ground diameter, and canopy width); conservation did not significantly change herb layer. The herb height and coverage were about 25 cm and 15%–20%, respectively. The conservation‐induced linear decline in herb density was from 0.30 to 0.15 indi/cm^2^ (Table A3 in Appendix S1). Forest types of succession from miscellaneous woods to larch climax forests, decreasing tree height and tree density. At the same time, declines in the shrub height but 2‐fold increases in the shrub coverage and 2.5‐fold increases in the shrub density were observed, together with the sparse herb (half herb density) and declining herb coverage and relative abundance (Table A3 in Appendix S1).

For plant diversity indices, conservation intensity from the low to the highest (core region) accompanied with the decline in tree richness, diversity, and evenness, with a middle‐peaked shrub richness, distribution evenness, and diversity at the buffer or experimental regions; the conservation also induced declines in herb richness about 1–5 species and herb distribution evenness, but without apparent changes in diversity indices (Table A3 in Appendix S1). From miscellaneous woods to larch climax forests, the decline of tree species richness was found from four species to less than two species. At the same time, diversity indices and distribution evenness also declined about 30% regarding that outside the Reserve. Differently, shrub richness, evenness, and diversity did not change obviously, and shrub species was around five species in the richness; herb species richness declined from 15 species to less than seven species, and diversity declined half compared with that outside the Reserve, and no significant changes were found in distribution evenness (0.6–0.7) (Table A3 in Appendix S1).

### 3.4 Association decoupling: RDA ordination and explaining factors for variations

**FIGURE 2:**
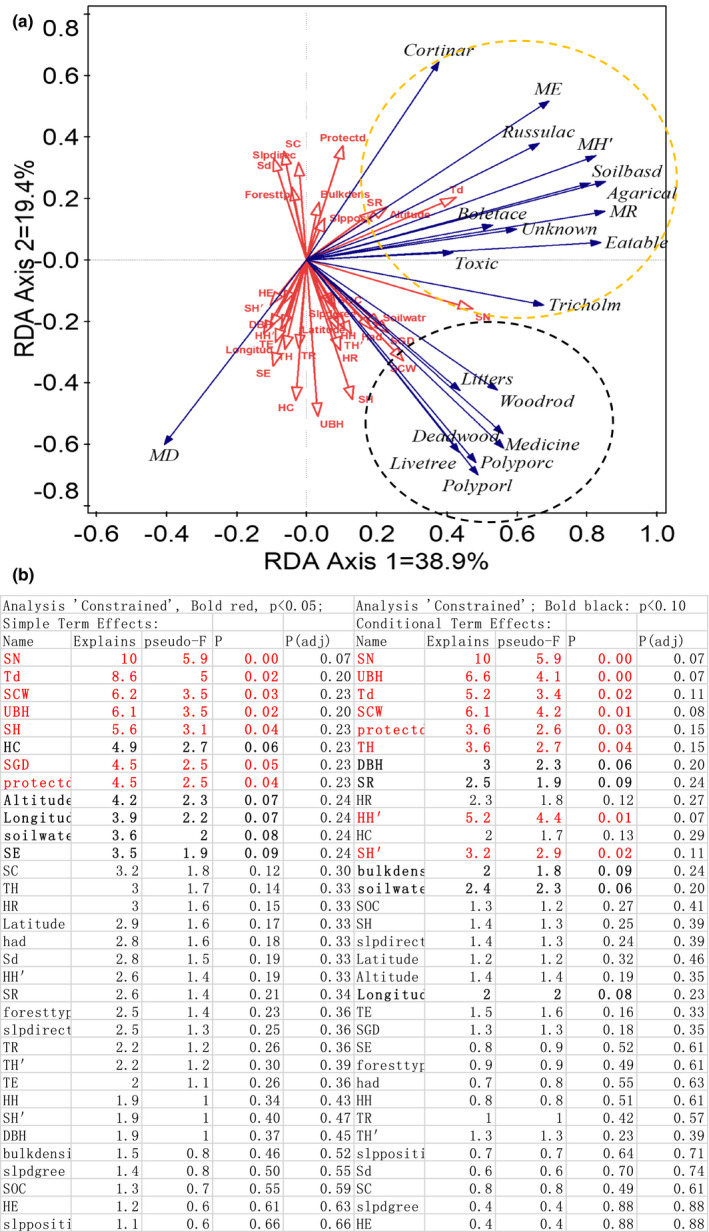
Association decoupling between macrofungi and various factors by RDA ordination. (a) RDA ordination map; (b) significant explaining factors from the simple term effect and the conditional term effect

We used RDA ordination to show the complex association between the macrofungal variation and other parameters surveyed (Figure 2a). In general, two groups could be identified from the ordination map. The first group (black dash cycle) included habitat function guilds of litters, living wood, and deadwood fungi and utilization functional guilds of medicine and wood‐rot macrofungi, Polyporales, and Polyporaceae. This group increased with soil N, shrub size (height, sh, and canopy width, scw), herb (richness, HR, and coverage, hc), and tree size (underbranch height, ubh). Still, it declined with increasing shrubs (coverage, sc, and density, sd) and increasing conservation intensity. The second group (yellow dash cycle) included macrofungal diversity, soil‐based macrofungi, macrofungal distribution evenness, edible macrofungi, Agaricales, Russulaceae, Tricholomataceae, Boletaceae, and Cortinariaceae. This group increased with tree density (td), soil N (SN), conservation intensity, and altitude (Figure 2a). Soil N positively aligned with the increase of all these two groups, while conservation contrarily affected two groups of macrofungi. For plant traits, trade‐off among different parameters was found in their association with macrofungal variation, for example, shrub density and shrub size negatively associated with the first group macrofungi, but almost no relations to the second group (Figure 2a).

The statistically significant parameters explaining the macrofungal variation are also listed in Figure 2b. The most potent explaining parameter was soil N, defining a total of 10% of the variation (*p* < .01). Tree (density, height, and underbranch height) explained 3.6%–8.6% (*p* < .05), shrub (canopy width, diameter, height, diversity) explained 3.2%–6.1% of the variation, and herb (richness and diversity) explained 2.3%–5.2%. Conservation measures could also explain the variation significantly, such as conservation intensity (3.6%–4.5%) and the position (latitude, longitude, and altitude; 2%–4.2%). Both simple term effects and conditional term effects showed similar results (Figure 2b).

### 3.5 Conservation pathway: SEM analysis

**FIGURE 3:**
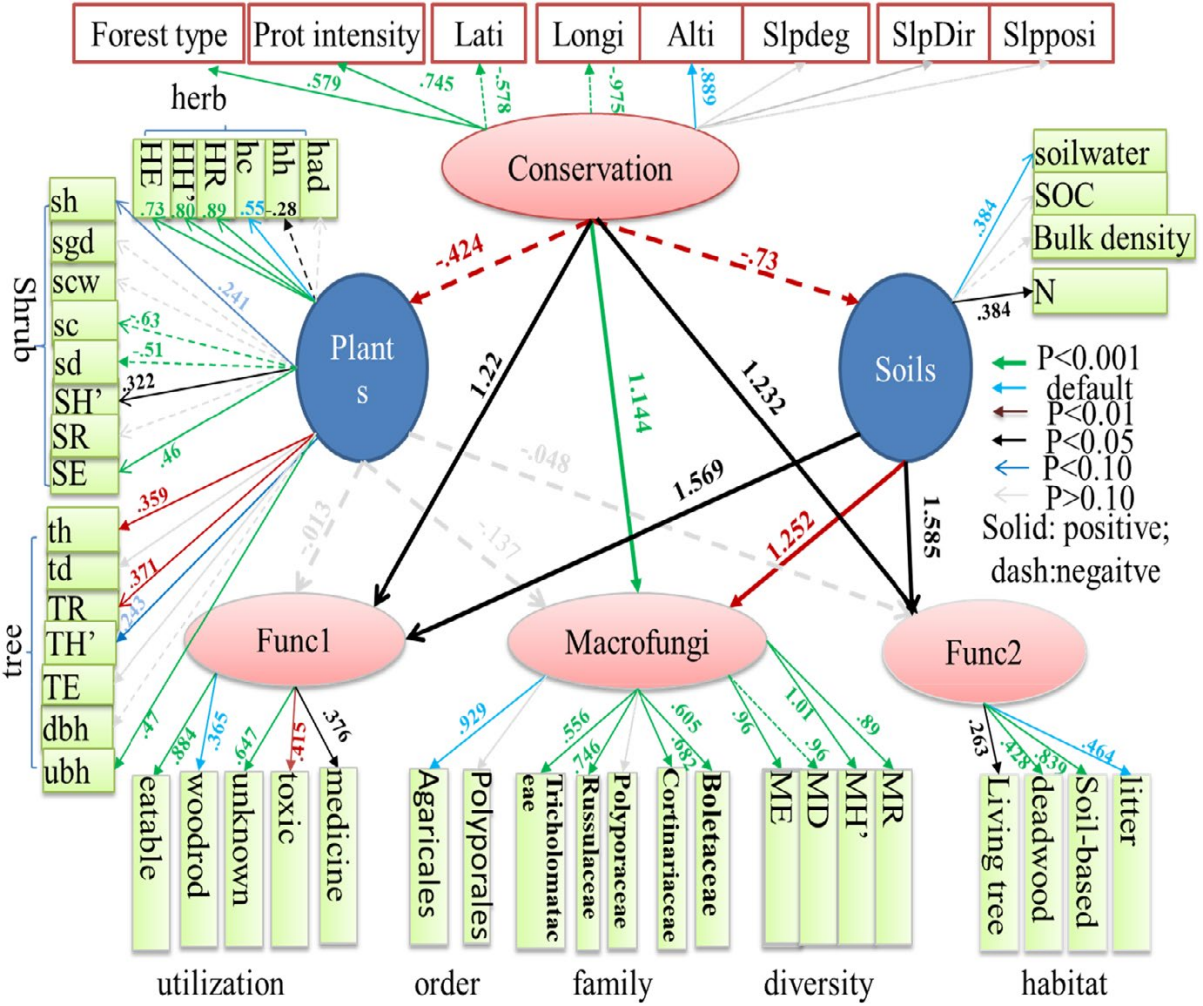
SEM analysis of the conservation effects on macrofungi (diversity and functional groups) and possible indirect pathways from soil and plants. All coefficients among latent variables (ellipse) and the coefficients (*p* < .10) were on the figure

We tried to separate the effects of conservation measures (site selection, forest type selection, conservation intensity) on macrofungi directly or indirectly from soils or plants by SEM analysis (Figure 3). In the conservation measures, both site selection (latitude, longitude, and altitude, rather than slope features), conservation intensity, and forest type selection significantly contributed to the latent variable of conservation (*p* < .001). Moreover, the coefficients of site selection (longitude and altitude 0.889–0.975) were 20%–30% higher than conservation intensity (0.745) and 50%–70% higher than forest type selection (0.579), indicating that the location of the Reserve is essential for the conservation effects from the reserve construction (Figure 3).

All these conservation measures could affect soils, macrofungi, and plants (*p* < .05). For macrofungi, the coefficients for functional guilds (1.22–1.23) and diversity and taxonomy (1.144) were about 3‐fold higher than the coefficient for plants (−0.424) and 1.6‐fold higher than that for soils (−0.73). In detail, selection of forest type tending to climax larch forests, with the higher conservation intensity, and Reserve location more in southeast part (low longitude and latitude) at the higher altitudes could improve the habitat and utilization guilds. Macrofungal diversity and taxonomy conservation accompany lower soil N and plant traits (Figure 3). Conservation's adverse effects on plants and soils were cross‐confirmed by the observation in Table A3 in Appendix S1 and confirmed by the RDA ordination (Figure 2).

Comparison of conservation measures and plants, soils gave the strongest influences on macrofungi (coefficient 1.3–1.6, *p* < .05), followed by conservation measures (coefficient 1.1–1.2, *p* < .05) and the lowest effects was from plants (−0.01 to −0.14, *p* > .10). Moreover, conservation could also indirectly affected macrofungi via soils (−0.91 to −1.16, *p* < .05), and this indirect impact offset the direct conservation effects largely (coefficients 1.1–1.2). The indirect effect from plants was positive but rather smaller (coefficients, 0.00–0.06) (Figure 3).

**FIGURE 4:**
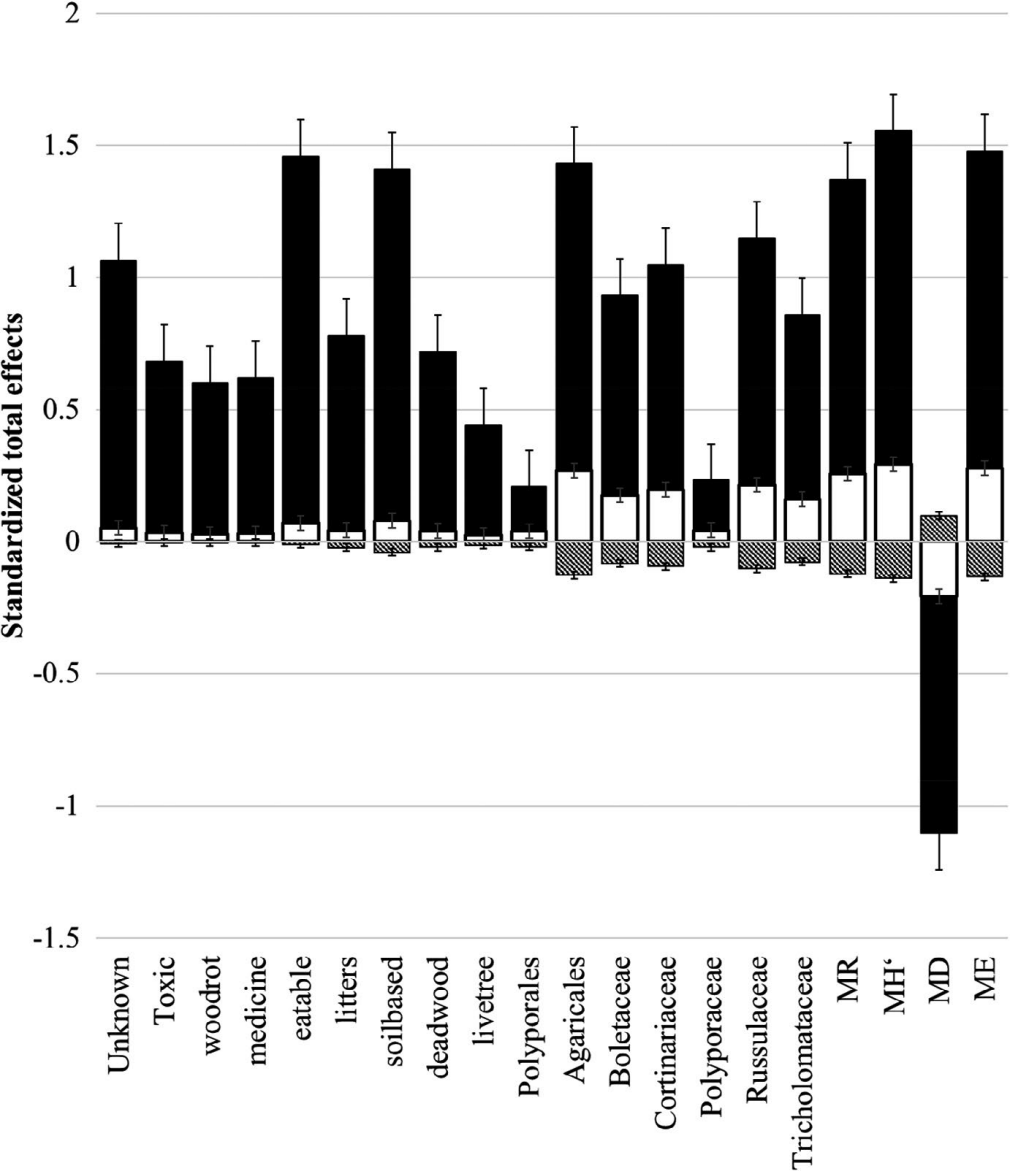
SEM‐derived effects from conservation, soil matrix, and plants on macrofungal diversity, taxonomic group, function groups of utilization, and habitats. The line column is the effect from plants, the black column is the effect from soils, and the white column is conservation

Figure 4 showed how many differences of conservation, soils, and plants on all 20 macrofungal parameters. Soil's effect on macrofungi was usually positive at 0.5–1.5, and 4‐ to 10‐fold much more extensive than those from plants, 2‐ to 5‐fold higher than those from conservation measures (maximum coefficient at 0.3). Almost all the effects on macrofungi (function group related to utilization and habitat) were from soils (relative contribution from soils >95%); however, to other macrofungal taxon and diversity parameters, conservation's effects become essential (21%), and plants usually showed the trade‐off effects for the conservation with negative contribution (10%) (Figure 4; Figure A1 in Appendix S1).

### 3.6 Effect size comparison: conservation and forest type

**FIGURE 5:**
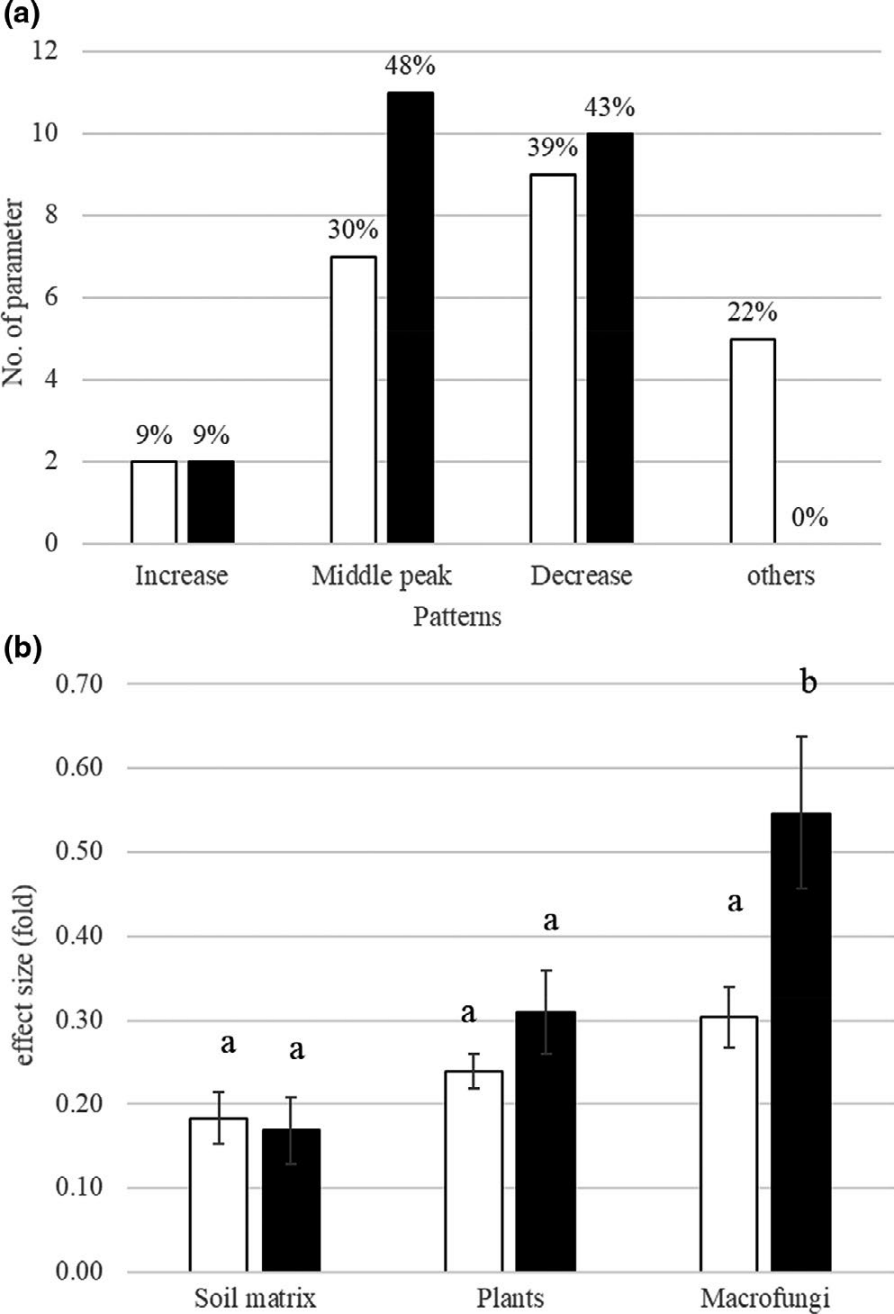
Impacts of conservation and forest type on macrofungi (a), and effect size comparison of macrofungi regarding their influences on soils and plants (b). Impacting patterns for an increase, middle‐peak, decrease, and others mean that from low conservation intensity to high intensity, from miscellaneous wood forest to climax forest, the macrofungal parameter increased, peaked at the middle stage, decreased or other changes except above three. The white column is conservation‐related parameters, and the black column is the forest‐type‐related parameters

Patterns of conservation and forest‐type impacting macrofungi were characterized as increased, middle‐peak, and decreased with protection or forward succession (Figure 5a). Conservation and forest‐type largely shaped macrofungal diversity. 69% of tested macrofungal traits declined with the conservation intensities or peaked at the middle conservation region (buffer‐experimental region of the Reserve). During forest succession from miscellaneous woods, birch‐larch forests to climax larch forests, 91% of macrofungal traits declined or peaked in the middle stage of birch‐larch forests.

The effect size comparison of macrofungi regarding their influences on soils and plants is shown in Figure 5b. Effect sizes for soil matrix from conservation and forest type alternations were 17%–18% of the controls. In the case of plants, the effect sizes were 24%–31% of the control. Much larger effect sizes were found in macrofungi. The effect sizes averaged 55% in different forest types and indifferent conservation regions; the effect sizes averaged 30%.

## 4 DISCUSSION

Forests in Daxinganling Mts in China are the southernmost boreal forests in Asia continents (Zhou, 1997). This region is characterized by larch climax vegetation with frequent fire disturbance (Xu et al., 1997). Hefty timber harvest has occurred in this region in history, which has primarily reduced tree size in this region (Wang et al., 2021). As shown in this paper, timber even in the Reserve is smaller than 14 cm in DBH. This has resulted in the implementation of NFPP in China to ban timber harvest in this region to recover the natural forests and engagement in nontimber forest products (NTFPs) business activities (Zhu et al., 2017). Timber harvest residents have to change their lives to understory resources, including wild macrofungus collection (Wu et al., 2019). A local forest management system could improve household livelihood and reduce area poverty by enabling efficient use of macrofungi to generate more outstanding sales (Zhu et al., 2019). This background put the boreal forests in a different situation from those in Canada, Europe, and Russia and add new data for boreal forest conservations. Our data in this paper are a case.

### 4.1 Macrofungal conservation and forest‐type differences strongly interacted with the soil matrix

**FIGURE 6:**
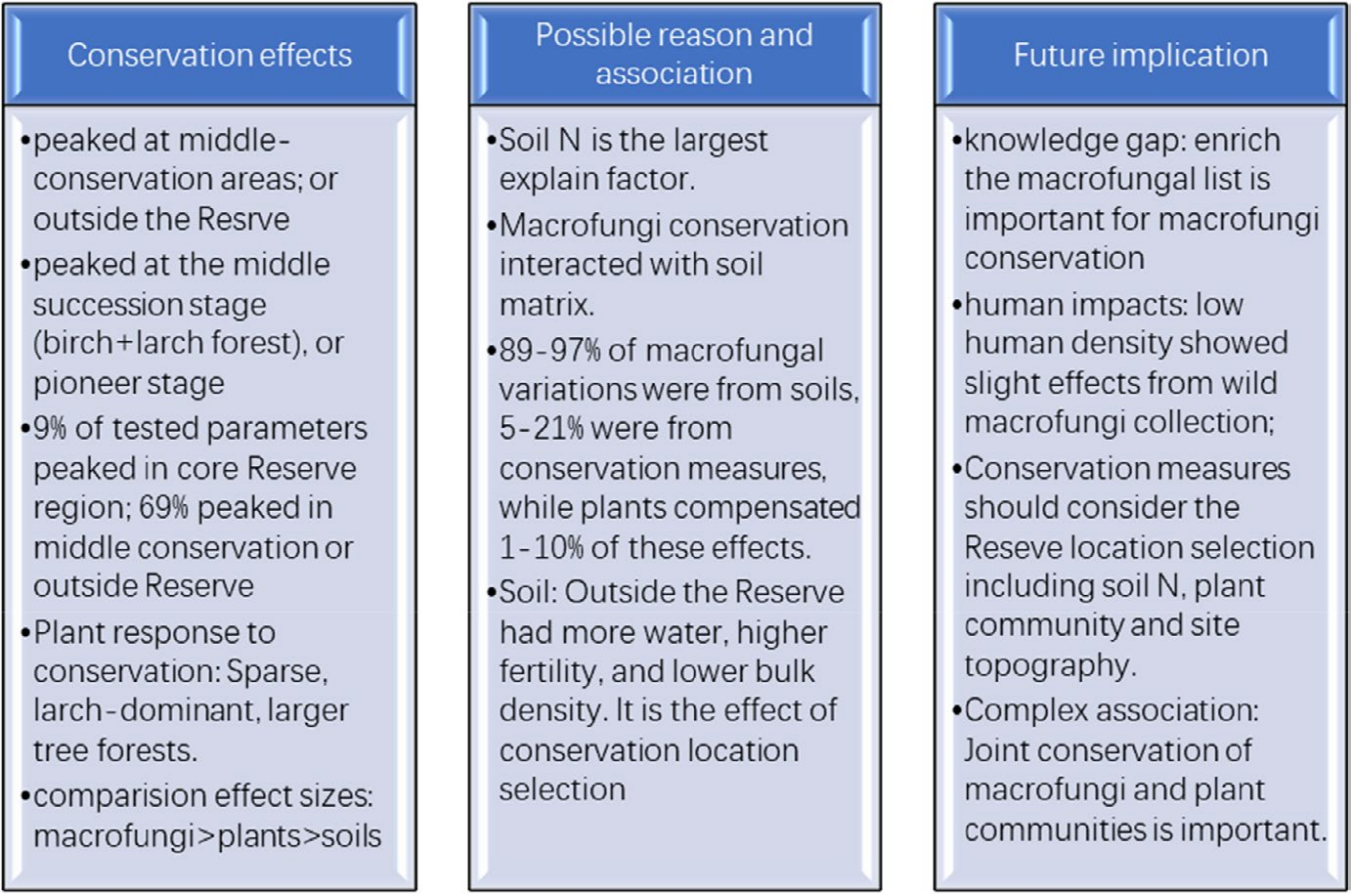
Conservation effects, possible reasons, and future implications derived from this study

Conservation changed macrofungi composition, and in most cases, macrofungal species richness and diversity indices did not peak at the core region of the Reserve. Peak values were generally observed in the middle‐conservated areas (Figure 6). For example, edible macrofungi, medicinal macrofungi, soil–habitat macrofungi, macrofungi richness index, Shannon–Wiener index and evenness index, Agaricales, and Russulaceae peaked at buffer or experimental regions. The linear decreasing pattern was also observed in some parameters, such as peak values at outside Reserve was found in living‐wood macrofungi, Simpson index, plot‐average order number, and Polyporaceae, Polyporales. After implementing NFPP, the macrofungal collection has become a necessary income for local people, and many scientists caution the possible decreases of macrofungal diversity from these human activities. In our study, we do not find sharp declines in the un‐conservated region outside the Reserve. It means that it is no need to worry about human collection‐related declines in macrofungi richness and abundance in this region.

The Natural Reserve aims in boreal forests to protect the climax vegetation of larch forests and biological resources in this forest. This region's heavy timber harvest has resulted in secondary miscellaneous wood forests and birch + larch mixed forests (Zhong, 2021). Macrofungi are usually strongly associated with forest types (Bau et al., 2019; Shao & Xiang, 1997). From secondary forests to climax larch forests, this forwarding succession accompanied with the abundance declines in medicinal, wood‐rot, toxic, and unknown‐function macrofungi, deadwood‐, living wood‐, and litter‐habitat macrofungi, as well as Polyporales, Polyporaceae, and Tricholomataceae. However, birch + larch mixed forests showed a higher abundance of edible macrofungi, macrofungi richness index, and Agaricales and Russulaceae.

As mentioned above, forest‐type and conservation measures could powerfully shape macrofungi composition (Figure 6). Much more significant macrofungal changes were found than the changes in soil properties and plant traits in conservation and forest types. In the case of forest types‐related macrofungi variation, macrofungi showed 1.5‐ to 3‐fold higher changes than those for soils and plants (*p* < .05). In the case conservation effects, macrofungal changes were 1.3‐ to 1.7‐fold higher than those of soils and plants. More studies have focused on trees, shrubs, and herbs in taxon and diversity indices (Zhang et al., 2018). In this study, we also found conservation can induce the observation of more giant trees, sparse density by fewer tree species, together with shorter but higher coverage of shrubs and much fewer herb species. In a temperate forest 900 km southeastern to this study, the conservation effect for plants is characterized as significant increases in tree sizes with great changing (plus or minus) species abundances (Wang et al., 2020). The boreal forest has also been highlighted for their frozen soils for a large amount of carbon and greenhouse gas storage (Huang et al., 2018; Li et al., 2018), and forest biomass changes during the frequent wildfire (Chang et al., 2008; Chao et al., 2017; Gao et al., 2018; Hu et al., 2018; Li et al., 2004). With the implementation of NFPP in China (Zhang et al., 2000), the wild mushroom economy has been encouraged by local governments (Bau & Li, 2010; Minter et al., 2012; Wu et al., 2019). Our finding strongly suggested that macrofungi's much more significant effect should also be evaluated in biodiversity evaluations in future boreal forest evaluations.

We also found the reason for such significant variations in macrofungi in the RDA ordination and variation partitioning. Soil matrix, rather than plants, gave the most considerable explaining power for the observed macrofungal variations (Figure 6). Some scientists argued forest‐related traits were essential for macrofungal conservation. It is recommended surrogates for macrofungal conservation by using mosses (Mcmullan‐fisher et al., 2010) and tree species richness (Li et al., 2021; Schmit et al., 2005). Declines of decaying woods (Rydin et al., 1997) and old growth‐like forests (Brazee et al., 2014; Heine et al., 2019), as well as habitat degradation (rather than landscape fragmentation) (Brown et al., 2006), broadly threaten fungal diversity and conservation. Recent studies argued that soil matrix and site selection are essential for preservation. This importance has been manifested by the soil nutrient–fungi relations (Hobbie & Hobbie, 2008), soil moisture and fertility determining roles (Rydin et al., 1997), ecosystem moisture and nitrogen role in macrofungal diversity (Trudell & Edmonds, 2004), long‐term nitrogen‐mediated changes in fruiting ectomycorrhizal species (Tarvainen et al., 2003), and the high fidelity in macrofungal species composition along natural N gradients and N deposition in forests (Cox et al., 2011; Kjøller et al., 2012; Kranabetter et al., 2009; Rühling & Tyler, 1991). In boreal forests in Europe, physiochemical soil properties are vitally important for plant community succession after fire disturbance (Gustafsson et al., 2021), while legacy effects (the previous land uses and forest management) are also crucial for understory species changes (Gustafsson et al., 2021).

Soils outside the Reserve had more water, higher N and SOC, and lower bulk density. Such grounds usually prefer miscellaneous wood forest distribution (the earlier stage of forest succession after a fire). Owing to the prolonged soil changes during conservation, the difference in soil should reflect the status for the original location features of the Nature Reserve. Both RDA ordination and SEM analysis confirmed that this kind of soil difference gives a determining role in explaining macrofungal variations. Soil N is the most potent factor with the highest explaining percentage of 10%, positively related to almost all the macrofungal parameters (Figure 2). A standardized total effect comparison showed that 89%–97% was from soils. In contrast, plants showed adverse effects <10% (Figure 4).

Furthermore, the macrofungal collection tends only to affect edible mushrooms in general. However, our data do not find any differences in edible macrofungi at the conservation gradients, similar to other functional guilds. A possible reason for the determining role for macrofungi variations is soil matrix rather than human collections. Unlike some regions in south China with dense human populations, human disturbance is the main threat for wild macrofungi conservation (Mysological‐Society‐of‐China, 2016).

### 4.2 Implications: knowledge gap, human impacts, and complex association

Firstly, our data enrich the macrofungal lists of Huzhong Nature Reserve, and more detailed data will support well conservation and utilization in the boreal forest region in China (Figure 6). Comparison with the macrofungi list in this Reserve, our data have updated local macrofungal list (Zhuang, 2010); for whole, Daxinganling Mts showed 452 species (https://wenku.baidu.com/view/b5596dd176232f60ddccda38376baf1ffc4fe3e0.html). Deng also listed macrofungi in Daxinganling Mts in 3 books (Deng, 2010; Deng & Sun, 2010; Deng & Wang, 2010). As shown in Table A4 in Appendix S1, our list also updated these lists. This update of history indicates a significant gap for fundamental information shortage of wild macrofungi. There need more works to recognize the resource basic data for future utilization and effective conservation (Bau et al., 2019; Du et al., 2017). This data shortage is not only in China (Li et al., 2021) but also in the whole world (Minter et al., 2012) and needs to be highlighted in future research.

Secondly, human impacts from wild macrofungi collection did not primarily affect macrofungi composition and diversity in the boreal forest region. At the same time, conservation measures should fully consider the proper site, soil matrix, and plants. The human population density in the boreal forest is much lower than temperate forests in China (Figure 6). In this study, the population density is 60,000 people/km^2^. In the temperate forest region (Liangshui, Figure 1), the population density is 190,000 people/km^2^. The macrofungal conservation effect in this region is 30%, which is much lower than the conservation effect in temperate forests (60%–140%) (data not shown here). High populations around the Reserve could enlarge the conservation effects, possibly due to timber‐harvest‐ban policy turning local timber‐dependent economics to understory‐resource dependence including mushroom collection (Bau & Li, 2010; Mysological‐Society‐of‐China, 2016; Wang et al., 2012; Wu et al., 2019). In the future, macrofungi conservation should be fully considered natural conditions (such as soil matrix importance in this paper) and social development, including population density.

In the case of the macrofungi conservation, we should fully consider the complex association and conservation measures (location, forest types, topography), soil properties (N), and plant traits are top parameters for taking into accounts. The priority region selection of protected lands is vital for conserving more species at the least cost (Myers et al., 2000). These conservation effects could be compromised by the indirect impact from soils (Figures 3 and 4). In practices in China, the protected land is usually placed in a remote region far away from human arrival, with significantly fewer concerns on soil properties. In this study, Huzhong Reserve is 70km away from the nearest town, about 200 m higher than the outside Reserve in altitude, 2‐ to 5‐degree steeper of the slopes than the outside Reserve (Table A3 in Appendix S1). Most macrofungal parameters were positively related to soil N. As the most potent explaining factor, soil N could explain 10% of total macrofungal variations (Figure 2a). The nitrogen economy of boreal conifers has been highlighted for genecology and climate change mitigation (Kranabetter, 2014) and macrofungal linkage to N forms in soils (Koide et al., 2011). Our results showed that the boreal forest soil matrix is the most important factor explaining the macrofungal variations, and future location selection for nature reserve should consider more.

## 5 CONCLUSION

Macrofungi are essential for boreal forest function and resident livelihood in China. Conservation and forest types significantly shape the macrofungal composition. In most cases (78% of the parameters), peak values were generally found in the middle‐protected region, middle‐successional stage, or outside the Reserve and early successional stage. These macrofungal changes were complexly associated with the locations, soils matrix, and plant traits. Soil properties (particularly soil N) are the most potent explaining factors for the macrofungal variation. Our data support the conservation of macrofungi together with plants in boreal forests.

## CONFLICT OF INTEREST

The authors declare that they have no known competing financial interests or personal relationships that could have appeared to influence the work reported in this paper.

## AUTHOR CONTRIBUTIONS

**Wenjie Wang:** Conceptualization (lead); data curation (equal); formal analysis (equal); funding acquisition (lead); supervision (lead); writing‐original draft (lead); writing‐review and editing (lead). **Jingxue Sun:** Data curation (equal); writing‐original draft (equal); writing‐review and editing (equal). **Zhaoliang Zhong:** Data curation (equal); investigation (equal). **Lu Xiao:** Investigation (equal); software (equal); writing‐review and editing (equal). **Yuanyuan Wang:** Data curation (equal); methodology (equal); writing‐review and editing (equal). **Huimei Wang:** Conceptualization (equal); data curation (equal); writing‐review and editing (equal).

## Supporting information

Appendix S1

## Data Availability

Raw data of the topographic, soil properties, forest plant composition data, and macrofungi data can be found at Dryad: https://doi.org/10.5061/dryad.5mkkwh768.
