# Supplementary material for: Relating macrofungal diversity and forest characteristics in boreal forests in China: Conservation effects, inter‐forest‐type variations, and association decoupling
Source: Ecol Evol. 2021 Aug 27;11(19):13268–82. doi: 10.1002/ece3.8049 (PMC8495802; doi:10.1002/ece3.8049)
Supplement: Supplementary file 1 — Appendix S1 [file ECE3-11-13268-s001.docx]

**Online Appendix**


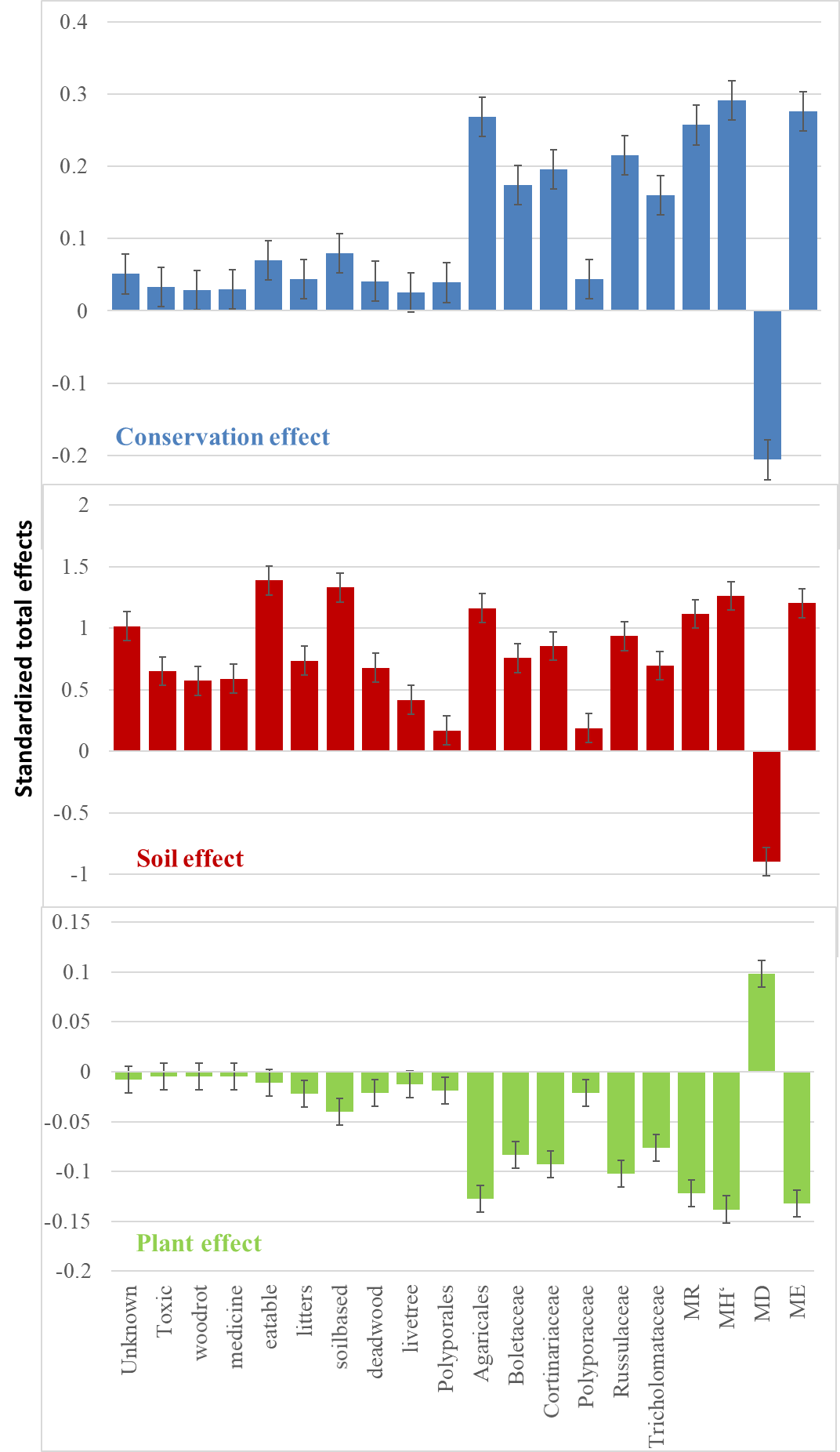


Fig. A1. Raw data of the SEM-derived effects from conservation, soil matrix, and plants on macrofungal diversity, taxonomic group, function groups of utilization, and habitats. Conservation effect could be predicted by the following equations (Conservation=0.89alti-0.97longi-0.58lati+0.75prot+0.58type). Soil effects could be predicted by the following equations (Soils=0.384N+0.384water-0.188bulkdensity+0.211SOC). Plant effects could be predicted by the following equation of Plants=tree(0.36H+0.47UBH+0.37Rich) +Shrub(0.46Even+0.32diversity-0.63cover-0.51density) +herb(0.89rich+0.80diversity+0.73even-0.55cover).

**Table A1 Functional guilds and diversity indexes of macrofungi in different protection regions, and their effect sizes and changing pattern.**

| Classification | Conservation intensity from low to high | | | | Effect size  (fold) | Pattern |
| --- | --- | --- | --- | --- | --- | --- |
|  | 1OutsideR | 2Buffer | 3Experi | 4Core |  |  |
| Utilization functional guilds | | | | |  |  |
| Edible | **9.00ab** | **7.75a** | **12.54b** | **10.11ab** | 0.22 | Middle peak |
| Medicinal | **2.88ab** | **3.19b** | **2.00a** | **2.11ab** | 0.23 | Middle peak |
| Wood-rot | **0.96b** | **0.19a** | **0.46ab** | **1.06b** | **0.48** | Fluctuate |
| Toxic | 1.16a | 1.19a | 1.38a | 0.94a | 0.14 | No change |
| Unknown-function | 2.36a | 1.19a | 2.00a | 1.33a | 0.36 | No change |
| Habitat functional guilds |  |  |  |  |  |  |
| Deadwoods | **3.48b** | **1.31a** | **2.46ab** | **1.67a** | **0.48** | Decrease |
| Soil-based | **11.32a** | **10.19a** | **18.23b** | **12.50a** | **0.27** | Middle peak |
| Living woods | 1.64a | 1.19a | 0.92a | 1.06a | 0.36 | Decrease |
| Litter | 0.72a | 0.44a | 0.46a | 0.61a | 0.30 | No change |
| Diversity index | | | | |  |  |
| MR | **3.32a** | **3.38a** | **4.64b** | **3.32a** | 0.14 | Middle peak |
| MH’ | **2.59a** | **2.68a** | **3.97b** | **3.16ab** | 0.26 | Middle peak |
| MD | **0.82c** | **0.81c** | **0.73a** | **0.76b** | 0.07 | Decrease |
| ME | **0.98a** | **1.03ab** | **1.32c** | **1.16b** | 0.19 | Middle peak |
| Taxonomy order (2 main order; observation/plot average were listed) | | | |  |  |  |
| Agaricales | **123/12.5a** | **67/12.0a** | **80/18.7b** | **72/13.4ab** | 0.20 | Middle peak |
| Polyporales | 29/2.6a | 14/2.0a | 11/1.7a | 19/2.0a | 0.27 | Decrease |
| Total Order/Mean/plot | 8/0.32 | 3/0.23 | 4/0.25 | 2/0.11 | 0.39 | Decrease |
| Taxonomy family (5 prominent families; observation/plot average were listed) | | | |  |  |  |
| Cortinariaceae | **21/3.6a** | **14/3.6a** | **16/4.3ab** | **17/5.9b** | 0.28 | Increase |
| Boletaceae | **13/1.4a** | **11/1.9ab** | **11/2.2ab** | **9/2.6b** | 0.60 | Increase |
| Russulaceae | **20/1.88a** | **17/3.50a** | **19/5.69b** | **19/3.28a** | 1.21 | Middle peak |
| Polyporaceae | 26/2.5a | 12/1.9a | 10/1.6a | 17/1.7a | 0.31 | Decrease |
| Tricholomataceae | 38/3.6a | 19/2.4a | 17/3.5a | 17/2.4a | 0.23 | No change |
| Total family/Mean/plot | 21/0.84 | 13/1.00 | 13/0.81 | 12/0.67 | 0.14 | Middle peak |
| Total observation/Mean/plot | 159/6.36 | 83/6.38 | 94/5.88 | 91/5.06 | 0.09 | Decrease |

Note MR: macrofungal richness index, MH’: macrofungal Shannon-wiener index, MD: macrofungal Simpson index, ME: macrofungal Pielou evenness. In the same row, the different letters showed significant differences between the 2 data at p < 0.05. We also used bold font to highlight the significant differences in other conservation regions (*p* <0.05).

**Table A2 Functional guilds and diversity indexes of macrofungi in different forest types, and their effect sizes and changing pattern.**

| Group | forest types from pioneering to climax vegetation | | | Effect size  (fold) | Pattern (linear or peak) |
| --- | --- | --- | --- | --- | --- |
|  | 1Miscellaneous woods | 2Birch-larch forest | 3Larch forest |  |  |
| Utilization guilds | | | |  |  |
| Edible fungus | **6.79a** | **10.93b** | **9.77ab** | 0.52 | Middle peak |
| Medicinal fungus | **3.21b** | **2.86ab** | **2.07a** | 0.23 | Decrease |
| Wood-rot fungus | 0.93a | 0.68a | 0.67a | 0.27 | Decrease |
| Toxic fungus | 1.43a | 1.11a | 1.07a | 0.24 | Decrease |
| Unknown-function | 2.00a | 1.89a | 1.57a | 0.14 | Decrease |
| Habitat guilds |  |  |  |  |  |
| Soil-based | **8.14b** | **13.79a** | **13.60a** | 0.68 | Middle peak |
| Deadwoods | **3.93b** | **2.21a** | **1.77a** | 0.49 | Decrease |
| Living woods | 1.57a | 1.43a | 0.97a | 0.24 | Decrease |
| Litters | 0.64a | 0.61a | 0.53a | 0.11 | Decrease |
| Diversity index | | | |  |  |
| MR | **2.85b** | **4.03a** | **3.47ab** | 0.32 | Middle peak |
| MH’ | **2.09a** | **3.33b** | **3.11b** | 0.54 | Middle peak |
| ME | **0.87b** | **1.17a** | **1.13a** | 0.32 | Middle peak |
| MD | **0.84b** | **0.77a** | **0.77a** | 0.08 | Decrease |
| Taxonomy order (top 2 main orders only; observation/plot average were listed) | | | | | |
| Agaricales | **81/9.64a** | **118/15.54b** | **94/14.0ab** | 0.53 | Middle peak |
| Polyporales | 24/2.86a | 22/2.29a | 21/1.73a | 0.30 | Decrease |
| Total Order/Mean/plot | 6/0.21 | 5/0.35 | 6/0.20 | 0.36 | Middle peak |
| Taxonomy family (top 5 prominent families only; observation/plot average were listed) | | | | | |
| Boletaceae | **9/1.00a** | **12/2.00b** | **11/2.40b** | 1.20 | Increase |
| Russulaceae | **14/1.86a** | **26/4.00b** | **20/3.27ab** | 0.95 | Middle peak |
| Cortinariaceae | **11/1.29a** | **23/4.89b** | **20/4.87b** | 2.78 | Increase |
| Tricholomataceae | 28/3.64a | 31/3.25a | 25/2.53a | 0.21 | Decrease |
| Polyporaceae | **22/2.71b** | **20/2.11ab** | **19/1.57a** | 0.32 | Decrease |
| Total family/Mean/plot | 17/0.61 | 19/1.35 | 16/0.53 | 0.67 | Middle peak |
| Total observation/Mean/plot | 111/3.96 | 145/10.36 | 119/3.96 | 0.81 | Middle peak |

Note MR: macrofungal richness index, MH’: macrofungal Shannon-wiener index, MD: macrofungal Simpson index, ME: macrofungal Pielou evenness. In the same row, the different letters showed significant differences between the 2 data at *p* < 0.05. We also used bold font to highlight the significant differences in different forest types (*p* <0.05).

**Table A3 Geo-topographic features and soil differences in different protected areas and forest types**

| Parameters | | Conservation intensity from low to high | | | | Forest types: succession low to high | | |
| --- | --- | --- | --- | --- | --- | --- | --- | --- |
|  |  | 1OutsideR | 2Buffer | 3Experi | 4Core region | 1Miscellaneous  woods | 2Birchlarch forest | 3Larch forest |
| Geotopographic factors | | | | | | | | |
| Longtitude | | 123.47c | 123.03b | 123.00ab | 122.88a | 123.61b | 123.02a | 123.04a |
| Latitude | | 51.95b | 51.73a | 51.78a | 51.71a | 51.92b | 51.77a | 51.79a |
| Altitude m | | 676.07a | 809.36b | 807.68b | 873.46b | 629.68b | 820.84a | 809.15a |
| Slope position | | 2.00a | 1.81a | 1.54a | 1.78a | 1.93a | 1.96a | 1.63a |
| Slope degree | | 9.47a | 8.54a | 5.52a | 11.32a | 10.19a | 8.63a | 8.83a |
| Slope direction | | 1.72a | 1.81a | 1.77a | 1.78a | 1.64a | 1.86a | 1.73a |
| Soil properties | | | | | | | | |
| Soil moisture (%) | | 46.19a | 30.73a | 35.83a | 29.96a | **56.97b** | **29.07a** | **34.66a** |
| Bulkdensity(g· cm^-3^) | | 0.89a | 0.94a | 0.99a | 0.99a | 0.87a | 1.01a | 0.91a |
| SOC (kg· m^-2^) | | 10.47a | 8.86a | 8.84a | 9.18a | 11.34b | 8.52a | 9.55a |
| N storage (kg· m^-2^) | | 0.42a | 0.29a | 0.39a | 0.34a | **0.46b** | **0.32a** | **0.36ab** |
| Individual size and density | | | | | | | | |
| Tree layer | TH (m) | **13.99b** | **12.23ab** | **11.33a** | **13.79ab** | **16.1b** | **11.8a** | **13.5c** |
|  | UBH m | 8.52a | 6.36ab | 5.16b | 7.97a | **10b** | **6a** | **7.1a** |
|  | DBH (cm) | **12.24a** | **12.51a** | **12.46a** | **16.07b** | 12.8a | 13.4a | 14.2a |
|  | Td(ind/m^2^) | 0.14a | 0.15a | 0.18a | 0.12a | 0.2a | 0.1a | 0.1a |
| Shrub layer | SH (m) | **0.64a** | **0.4b** | **0.39b** | **0.46ab** | 0.7a | 0.5a | 0.4a |
|  | SGD (cm) | 0.99a | 0.84a | 0.53a | 1.05a | 0.6a | 1a | 0.9a |
|  | SCW (cm) | 30.76a | 28.12a | 29.62a | 32.42a | 14a | 16a | 13a |
|  | SC (%) | **30.44a** | **51.78b** | **47.39ab** | **40.26ab** | **24.4b** | **43.5a** | **48.8a** |
|  | Sd(ind/m^2^) | 53.9a | 75.82a | 48.73a | 83.37a | **29.9a** | **66.2ab** | **80.7b** |
| Herb layer | HH(m) | 26.23a | 24.34a | 27.6a | 27.35a | 24.1a | 26.7a | 26.2a |
|  | HC (%) | 19.53a | 20.06a | 18.43a | 13.97a | 26a | 17.2a | 16.9a |
|  | HAD (%) | 12.04a | 8.41a | 12.03a | 10.33a | 44.6a | 31.2a | 30.1a |
|  | Hd(ind/cm^2^) | 0.3a | 0.18a | 0.18a | 0.14a | **0.42a** | **0.17b** | **0.2b** |
| Diversity characteristics | | | | | | | | |
| Tree layer | TR | **2.78b** | **1.88a** | **1.69a** | **2.06a** | **3.7b** | **2.3a** | **1.7c** |
|  | TH’ | 0.6a | 0.56a | 0.51a | 0.44a | **0.9b** | **0.6a** | **0.2c** |
|  | TE | 0.16a | 0.12a | 0.1a | 0.1a | **0.7a** | **0.8a** | **0.2b** |
| Shrub layer | SR | **4.24a** | **5a** | **6.46b** | **5a** | 5.2a | 5.0a | 5.2a |
|  | SH’ | **0.61ab** | **0.53a** | **0.82b** | **0.4a** | 0.8a | 0.5a | 0.6a |
|  | SE | 0.14a | 0.12a | 0.16a | 0.07a | 0.5a | 0.3a | 0.4a |
| Herb layer | HR | **10.42b** | **9.13ab** | **5.15a** | **7.33ab** | **15.1b** | **7.4a** | **6.5a** |
|  | HH’ | 1.2a | 1.2a | 0.78a | 0.98a | **1.8b** | **1.1a** | **1a** |
|  | HE | **0.22ab** | **0.27a** | **0.16b** | **0.19ab** | 0.7a | 0.6a | 0.6a |

Note： The different letters indicate significant differences between these two treatments (*p* < 0.05). We also used bold font to highlight the significant differences in different forest types and conservation regions (*p* <0.05).

**Table A4 the macrofungi species list of Liangshui National Nature Reserve and adjacencies (IUCN, +:VU, vulnerable, and all others are LC,** **Least Concern and DD, Data deficient; *: supplement for Deng XL 2010a, b, c; #, update for Zhuang HX 2010)**

**Basidiomycota**

1. *Oudemansiella mucida* summer-autumn on stump or deadwood in broadleaf forest, clumped sometimes separated. Eatable.
2. *# *Oudemansiella brunneomarginata* summer-autumn on stump or deadwood, clustered or separated. Eatable.
3. *# *Clitocybe phyllophila* common in forest land, clustered, sometimes clustered-like. Toxic.
4. *# *Clitocybe geotropa* autumn in forest land or grassland. Medicinal.
5. *Clitocybe maxima* summer-autumn on land or litters, clustered or separated. Eatable.
6. * *Clitocybe infundibuliformis* autumn in forest land or decomposed litters or on grassland. Eatable.
7. # *Clitocybe inversa* autumn in forest land, clustered or clumped. Eatable.
8. *# *Clitocybe odora* summer-autumn in decomposed litters, clustered or clumped. Eatable, Medicinal.
9. *# *Clitocybe candicans* summer-autumn in forest lands, clustered or clumped. Eatable.
10. *# *Pseudoclitocybe cyathiformis* summer-autumn in forest lands or dead wood, separated, clustered-like or clumped. Eatable.
11. * *Xeromphalina campanella* summer-autumn on deadwood or stump, a lot clustered. Medicinal.
12. * *Collybia acervata* Summer-autumn on broadleaf forest litters or rot-deadwood, clustered to clumped. Eatable.
13. *# *Collybia maculata* Summer-autumn on forest litters, rot-deadwood or lands, clustered to clumped-like. Eatable
14. *# *Collybia platyphylla* Summer-autumn on rot-deadwood, separated to clustered. Eatable, medicinal.
15. * *Collybia dryophila* Spring-summer in forest or forest-edge grassland or litters. Unknown-function for food or medicine.
16. *Collybia confluens* Summer-autumn on forest litters, clustered to clumped. Eatable,
17. *# *Tricholoma albobranneum* autumn on pine forest land, clumped or separated, sometimes in conifer-broadleaf mixed forest land. Eatable, medicinal.
18. *# *Tricholoma vaccinum* autumn on conifer forest lands, clumped or clustered. Eatable, mycorrhizal.
19. ^+^*# *Tricholoma matsutake* autumn on pine forest or broadleaf-conifer forest lands, separated or clumped. Eatable, mycorrhizal.
20. *# *Tricholoma fulvum* autumn on forest lands, Separated or clumped, sometimes clustered. Eatable, medicinal.
21. *# *Tricholoma album* summer-autumn on mixed forest lands, clumped or separated. Eatable.
22. # *Tricholomopsis rutilans* summer-autumn on conifer rot-wood or rot-stump, clumped or clustered. Toxic fungi.
23. *# *Tricholoma mongolicum* summer-autumn clumped on grassland forming mushroom cycles. Eatable, medicinal.
24. *Laccaria proxima* summer-autumn in forest lands, separated or clumped. Eatable, medicinal, mycorrhizal.
25. *Laccaria laccata* summer-autumn in broadleaf-Korean pine mixed forests, shaw forest litters. Separated or clumped. Eatable, medicinal.
26. # *Lyophyllum fumosum* autumn in forest land, in most cases in broadleaf or mixed forest land. Eatable, mycorrhizal.
27. *# *Lyophyllum cinerasceu*s autumn in forest land, clumped. Eatable, medicinal.
28. *Armillaria mellea* summer-autumn in different conifer or broadleaf tree’s stem base, root-base or down wood， clustered. Eatable, medicinal.
29. # *Armillariella cepistipes* summer-autumn on rot-woods, clumped, sometimes separated. Unknown-function for food or medicine.
30. *# *Melanoleuca strictipes* in mixed forest land or shrub grasslands, separated. Eatable.
31. *Lepista nuda* autumn in forest lands, clumped, sometimes clustered or separated. Eatable, medicinal.
32. # *Lepista sordida* summer-autumn in slope grasslands, clumped or clustered-like. Eatable.
33. *Lepista caespitosa* summer-autumn in forest lands. Eatable.
34. * *Lepista glaucocana* autumn in conifer and broadleaf forest lands, clumped. Eatable.
35. *Lepista personata* summer-autumn in forest lands, clumped or belts or mushroom cycles. Eatable, ectomycorrhizal.
36. *# *Mycena leptocephala* summer-autumn in forest short grasses, clumped. Unknown-function for food or medicine.
37. *# *Mycena alcalina* summer-autumn in forest lands, rot-woods or rot-litters, clustered. Eatable, medicinal.
38. *Mycena pura* summer-autumn in forest lands and rot-litters or rot-wood, clustered, clumped or separated. Eatable, medicinal.
39. *# *Mycena galericulata* summer-autumn in mixed forest rot-branches and litters or rot-woods, separated or clumped. Eatable, medicinal.
40. *# *Marasmius oreades* summer-autumn in grass lands, clumped and forming mushroom cycles, sometimes in forest lands. Eatable, medicinal.
41. *# *Marasmius scorodonius* summer-autumn on dead branches and litters, separated, clumped. Eatable.
42. *# *Clitopilus prunulus*spring summer at forest edge grasslands, separated or clumped. Eatable.
43. *# *Leucopaxillus giganteus*summer, autumn in forest grasslands, Separated or clumped. Eatable, medicinal.
44. *# *Leucopaxillus candidus* autumn in spruce forest land or grassland. Eatable.
45. # *Cystoderma cinnabarinum* summer-autumn in forest lands, separated. Eatable.
46. * *Cystoderma amianthinum* summer-autumn in conifer forest land. Eatable.
47. * *Lentinus lepideus* summer-autumn on conifer rot-woods, clustered. Eatable, medicinal.
48. *# *Hygrophorus turundus* summer-autumn in forest lands. Unknown-function for food or medicine.
49. # *Hygrophorus camarophyllus* summer-autumn in conifer forest lands. Eatable.
50. * *Hygrophorus puniceus* summer-autumn in conifer broadleaf forest lands, Separated or clumped. Eatable.
51. # *Hygrophorus chlorophanus* summer-autumn in forest lands or at forest edges and grasslands, clumped. Eatable.
52. * *Hygrophorus ceraceus* summer-autumn in forest lands, separated. Eatable.
53. *Hygrophorus lucorum* autumn in spruce etc. conifer forest lands, clumped or separated. Eatable, ectomycorrhizal
54. # *Hygrophorus leucophaeus* autumn in forest lands, separated or clumped. Eatable.
55. *Hygrophorus eburneus*, autumn in mixed forest lands, clumped or clustered. Eatable, mycorrhizal.
56. * *Hygrophorus ligatus* autumn in spruce, pine etc. conifer forest lands, clumped. Unknown-function for food or medicine.
57. # *Camarophyllus pratensis* spring-autumn in forest open lands, grasslands, separated or clumped. Eatable.
58. # *Gomphidius viscidus* in pine forest lands, separated or clumped. Eatable, medicinal.
59. *# *Chroogomphidius viscidus* clumped, separated or Separated in pine forest lands or grass-forest lands. Eatable, medicinal, ectomycorrhizal*.*
60. *# *Lepiota cristata*summer, autumn in conifer forest lands, grasslands, unknown-function for food or medicine.
61. # *Lepiota gracilenta*summer, autumn in forest lands and grasslands, separated or scattered. Eatable.
62. *# *Lepiota ventriosospora* summer-autumn in forest lands, forest edges and grasslands, separated or scattered. Eatable.
63. # *Lepiota clypeolaria*summer, autumn in mixed forest and grasslands. Eatable.
64. # *Agaricus silvaticus*summer, autumn in oak forest lands, clumped. Eatable.
65. # *Agaricus placomyces* autumn in forest lands and poplar root-bases, separated, clumped or clustered. Eatable, medicinal.
66. *Agaricus campestris* spring-autumn in grassland, roadsides, wild fields, forest lands etc. separated or clumped. Eatable.
67. *Agaricus arvensis* summer-autumn in spruce-fir forest or other conifer forest lands, separated or scattered. Eatable.
68. *# *Boletinus spectabilis* autumn in pine-spruce forest lands or rot-wood, rot branches and litters, separated or clumped. Eatable.
69. *# *Boletinus asiaticus* summer-autumn in scots pine, spruce, larch forest land between mosses or rot-wood arounds, Separated or clumped. Ectomycorrhizal.
70. *# *Boletinus paluster* summer-autumn in Korean pine, larch etc. conifer-broadleaf mixed forest land, sometimes on rot-woods, separated or clumped. Eatable, ectomycorrhizal.
71. *Boletinus cavipes* autumn in forest lands, clumped or clustered. Eatable, medicinal.
72. *Boletinus pictus* summer-autumn in pine forest or mixed forest lands, separated, clumped. Eatable.
73. *# *Boletus pallidus* summer-autumn in shaw forest lands, separated, clumped or clustered. Eatable. mycorrhizal.
74. *Boletus edulis* summer, autumn in forest lands, Separated or separated. Eatable.
75. *Suillus granulatus* summer-autumn in pine forest or mixed forest lands, separated, clumped or clustered. Eatable.
76. *Suillus flavus* in pine forest lands. Eatable.
77. *Suillus grevillei* autumn in larch forest lands, separated, clumped or clustered. Eatable.
78. *# *Suillus aeruginascens* summer-autumn in conifer forests, separated, clumped. Eatable.
79. *# *Xerocomus chrysenteron* summer-autumn in forest lands, separated, clumped. Eatable.
80. *Xerocomus badius* summer, autumn in conifer forest or broadleaf forest land, and grasslands, separated, clumped or clustered. Slight toxic, mycorrhizal.
81. *# *Tylopilus velatu*s in forest lands. Eatable.
82. *Leccinum scabrum* summer-autumn in broadleaf forest land, separated or scattered. Eatable.
83. *Leccinum crocipodium* in deciduous broadleaf forests, often in oak forest land. Eatable.
84. *Pholiota flammans* summer, autumn in conifer down woods, dead standing woods, logs or stumps. Eatable.
85. *# *Pholiota squarrosoides* autumn in broadleaf rot-wood or stumps. separated, clumped. Eatable.
86. *# *Stropharia rugosoannulata* spring-autumn in forest grasslands, roadsides, and horse dung, separated, clumped. Eatable.
87. *Kuehneromyces mutabilis* summer-autumn in deciduous forest stumps, or down wood, clustered. Eatable.
88. # *Cortinarius varius* autumn in conifer-broadleaf mixed forest land. Unknown-function for food or medicine.
89. *# *Cortinarius albovilaceus* autumn clumped in mixed forest lands. Eatable, ectomycorrhizal.
90. *# *Cortinarius obtusus* summer-autumn clumped in conifer broadleaf mixed forest lands. Eatable, mycorrhizal.
91. # *Cortinarius olivaceo-stramineus* autumn separated or clumped in forest lands. Unknown-function for food or medicine.
92. # *Cortinarius armillatus* summer-autumn separated or clumped in broadleaf-conifer forest lands. Eatable, ectomycorrhizal.
93. * *Cortinarius turmalis* autumn clumped in Korean pine etc. conifer forest land. Eatable.
94. *Cortinarius cinnamomeus* autumn clumped in fir forest lands. Eatable.
95. *# *Cortinarius claricolor* summer-autumn in mixed forest lands. Eatable.
96. *Cortinarius pholideus* summer-autumn clumped or clustered in spruce forest lands. Eatable, medicinal, ectomycorrhizal.
97. *Cortinarius multiformis* autumn clumped or separated in conifer forest or mixed forest lands. Eatable.
98. *# *Cortinarius malachius* summer-autumn in conifer-broadleaf mixed forest land. Mycorrhizal.
99. #*Cortinarius hinnuleus* summer-autumn in broadleaf forest lands, often clumped. Unknown-function for food or medicine, ectomycorrhizal
100. # *Cortinarius distans* autumn clumped or clustered in spruce, fir moss layers. Unknown-function for food and medicine.
101. *# *Cortinarius croceofolius* summer-autumn clumped in conifer-broadleaf mixed forest lands. Unknown-function for food and medicine.
102. *# *Cortinarius tenuipes* autumn clustered in conifer-broadleaf mixed forest land. Eatable.
103. *# *Cortinarius speciosissimus* summer-autumn in conifer forest lands. Toxic fungi.
104. *# *Cortinarius supferrugineus* conifer-broadleaf mixed forest lands, separated. Mycorrhizal.
105. *# *Cortinarius armeniacus* autumn in conifer-broadleaf mixed forest lands, clumped. Eatable, mycorrhizal.
106. *# *Cortinarius argentatus* autumn clumped or separated in spruce-fir forest and broadleaf forest lands. Unknown-function for food and medicine.
107. # *Cortinarius rufo-olivaceus* in spruce forest lands. Unknown-function for food and medicine.
108. * *Cortinarius purpurascens* autumn clumped or separated in mixed forest lands. Eatable, mycorrhizal.
109. *# *Leucocortinarias bulbiger* summer-autumn separated or scattered in Korean pine forest lands. Eatable.
110. *Gymnopilus aeruginosus*summer, autumn clumped or clustered in spruce-fir down woods. Unknown-function for food and medicine.
111. *Inocybe fastigiata* summer-autumn separated or clumped in broadleaf and pine forest lands. Toxic fungi.
112. *Inocybe caesariata* clumped in forest lands. Unknown-function for food and medicine.
113. *Rozites caperata* autumn separated or separated in forest lands. Eatable. Ectomycorrhizal.
114. * *Pleurotus ostreatus* Imbricated and clumped on broadleaf trees. Eatable.
115. *#*Pleurotus anserinus* summer-autumn clustered or imbricated on broadleaf-conifer forests- birch and oak dead woods. Eatable.
116. *Panus rudis* clumped, clustered or imbricated on broadleaf rot-woods. Eatable.
117. # *Panellus stypticus* summer-autumn in different broadleaf stumps or dead branches. Toxic, Medicinal.
118. *# *Amanita vaginata* spring-autumn separated or scattered in pine forest lands. Toxic. Ectomycorrhizal.
119. # *Amanita caesarea* summer-autumn separated or scattered in forest lands. Eatable. Ectomycorrhizal
120. # *Amanita muscaria* summer-autumn clumped in forest lands. Toxic fungi. Mycorrhizal.
121. *# *Amanita pantherina* clumped in pine forest, shaw lands. extreme toxic. pine, spruce’s mycorrhizal.
122. # *Rhodophyllus clypeatus* summer-autumn clumped or scattered in mixed forest lands. Eatable.
123. *# *Rhodophyllus speculu*s in broadleaf-conifer forest lands. Unknown-function for food and medicine.
124. # *Pluteus pellitus* summer-autumn on rot-woods, separated or clumped. Eatable.
125. # *Pluteus cervinus* spring-autumn separated or scattered, sometimes clumped in forest lands or on rot-woods. Eatable.
126. *# *Pl*uteus *depauperatus* autumn on rot-woods or wood chips. Unknown-function for food or medicine.
127. # *Psathyrella candolleana* summer-autumn separated or clustered in forest lands, wild fields, roadsides, also on rot stumps. Eatable.
128. *# *Psathyrella lactobrunnescens* summer-autumn clumped or clustered on rot-woods. Unknown-function for food or medicine.
129. * *Coprinus micaceus* spring-autumn clustered on broadleaf tree root base-around lands. Eatable.
130. * *Coprinopsis atramentaria* spring autumn clustered in forest lands, roadsides, and grasslands. Eatable.
131. * *Coprinus plicatilis* spring-autumn clumped on forest litters and branches. Unknown-function for food and medicine.
132. * *Panaeolus retirugis* spring-autumn on cow dung, horse dung or fertile soils, clumped. Toxic fungi.
133. *Hypholoma cinnabarinum* summer in forest slopes or on tree stump arounds. Toxic fungi.
134. * *Russula aeruginea* summer-autumn in pine forest or mixed forest land, separated or clumped. Eatable. Ectomycorrhizal.
135. *# *Russula lactea* summer-autumn separated or clumped in mixed forest lands. Eatable.
136. # *Russula integr*a summer-autumn separated or clumped in forest lands. Eatable, Ectomycorrhizal.
137. *Russula delica* summer-autumn in conifer forest or mixed forest lands. Eatable, Ectomycorrhizal.
138. *# *Russula flavida* summer-autumn separated or clumped in forest lands. Unknown-function for food and medicine.
139. # *Russula subdepallens* summer-autumn clumped in forest lands. Eatable.
140. # *Russula atropurpureaa* summer-autumn separated or clumped in forest lands. Eatable, Ectomycorrhizal.
141. * *Russula vinosa* summer-autumn clumped in broadleaf forest lands. Eatable, medicinal.
142. * *Russula cyanoxantha* summer-autumn scattered or clumped in broadleaf forest lands. Eatable, Ectomycorrhizal.
143. * *Russula aurata* summer-autumn separated or clumped in mixed forest lands. Eatable, Ectomycorrhizal.
144. *Russula lutea* summer-autumn scattered or clumped in conifer, broadleaf forest lands. Eatable, Ectomycorrhizal.
145. *Russula foetens* summer-autumn clumped or scattered in pine forest or broadleaf forest lands. Eatable, medicinal, ectomycorrhizal
146. *Russula emetica* summer-autumn clumped or scattered in forest lands. Medicinal. Ectomycorrhizal.
147. # *Russula olivacea* summer, autumn in forest lands. Eatable, Ectomycorrhizal.
148. *# *Russula patazurea* in broadleaf forest lands. Eatable, Ectomycorrhizal.
149. * *Russula mariae* summer-autumn separated or clumped in broadleaf forest lands. Eatable, Ectomycorrhizal.
150. *Russula decolorans* summer-autumn separated or clumped in pine forest lands. Eatable, Ectomycorrhizal.
151. *# *Russula sanguinea* summer-autumn clumped in conifer forest lands. Ectomycorrhizal.
152. *Russula ochroleuca* summer, autumn in moist birch or pine forest lands. Ectomycorrhizal.
153. *Russula paludosa* summer-autumn scattered or clumped in conifer forest or mixed forest land. Eatable.
154. *# *Russula luteolacta* summer-autumn in forest lands, scattered or clumped. Toxic fungi.
155. *Lactarius uvidus* summer-autumn separated or clumped in forest lands. Unknown-function for food and medicine.
156. *Lactarius zonarius* summer-autumn clumped or scattered in broadleaf-conifer forest and conifer forest lands. Eatable.
157. *Lactarius vellereus* summer-autumn separated or clumped in shaw lands. Eatable. Ectomycorrhizal.
158. *Lactarius deliciosus* summer-autumn separated or clumped in pine forest lands. Eatable. Mycorrhizal.
159. *# *Lactarius sanguifluus* summer-autumn separated or clumped in conifer forest lands. Eatable. Mycorrhizal
160. *# *Lactarius blennius* summer-autumn separated or clumped in broadleaf forest lands. Eatable. Ectomycorrhizal.
161. *Lactarius piperatuss* summer-autumn clumped or scattered in broadleaf-conifer forest lands. Eatable. Mycorrhizal.
162. *# *Lactarius torminosus* summer-autumn in forest lands, separated or scattered. Toxic fungi. Ectomycorrhizal
163. # *Lactarius hygroporoides* summer-autumn in shaw lands, separated or clumped. Eatable.
164. * *Fomes fomentarius* often on birch etc. broadleaf dead standing woods. Medicinal.
165. # *Gloeophyllum saepiarium* clumped on spruce, larch down woods. Medicinal.
166. *Ganoderma applanatum* on broadleaf dead standing woods, down woods, and stumps. Medicinal.
167. * *Ganoderma lucidum* on broadleaf stumps, sometimes on conifer stem bases. Medicinal, Eatable
168. *Ganoderma tsugae* on pine stem base or roots. Medicinal.
169. *Phellinus pini* on conifer living trees. Medicinal.
170. *Phellinus igniarius* on broadleaf stumps, stems or down woods. Medicinal
171. *Phellinus linteus* on poplar, oak, springa’s dead standing woods or living stems. Medicinal.
172. *Lenzites tricolor* on broadleaf rot-woods, sometimes on pine dead standing woods or down woods. often side-growth or imbricated. Medicinal.
173. # *Lenzites betulina* summer-autumn on broadleaf rot-woods, imbricated, sometimes also on spruce, fir etc. conifer rot woods. Medicinal.
174. # *Coriolus versicolor* on different broadleaf stumps, down woods, and dead branches. Medicinal.
175. #*Coriolus hirsutus* on poplar, Salix etc. broadleaf living stems, dead standing stems and branches or stumps. Medicinal.
176. *Corio*lus *unicolor* on birch, poplar etc. stumps, dead standing stems and down woods. Medicinal.
177. *Tyromyces fissilis* on broadleaf down woods. Unknown-function for food or medicine.
178. # *Tyromyces amygdalinus* summer-autumn on tree stems. Unknown-function for food or medicine.
179. *Piptoporus betulinus* on Betula genus stems. Young eatable, medicinal.
180. * *Pycnoporus coccineus* summer-autumn clumped or imbricated on oak, acer, poplar, Salix, etc. broadleaf dead woods, or pine, spruce dead woods. Medicinal.
181. * *Polyporus badius* summer-autumn separated or clustered on broadleaf rot-woods. Unknown-function for food or medicine.
182. #*Polyporus giganteus* summer-autumn in broadleaf stump-around lands. young eatable, medicinal.
183. *Bjerkandera fumosa* on spruce, birch etc. stumps, dead standing woods, down woods, imbricated. Medicinal.
184. *Bjerkandera adusta* on broadleaf down woods and dead standing woods. Medicinal.
185. *Ischnoderma resinosum* on spruce, Korean pine, elm’s living tree base, down wood, or dead standing tree. Eatable, medicinal.
186. # *Trametes corrugata* on broadleaf dead standing trees or down wood. Medicinal.
187. *Trametes orientalis* on broadleaf dead standing trees and rot-wood or crosstie wood. Medicinal.
188. # *Fomitopsis ulmaria* on elm etc. broadleaf stem base or down wood. Medicinal.
189. *Fomitopsis pinicola* on spruce, larch, Korean pine, birch’s down woods, dead standing tree, and stump as well as log. Medicinal.
190. *# *Daedaleopsis rubescens* on poplar, birch etc. broadleaf living stem, down woods, dead branches. Unknown-function for food or medicine.
191. *Laetiporus sulphureus* on Salix, spruce-like living stem, dead standing woods. Eatable.
192. *Hirschioporus abietinus* on spruce, larch dead standing trees, down woods or dead branches. Medicinal.
193. *Hericium coralloides* autumn on fir, spruce’s down rot-wood or stump or wood hole. Eatable.
194. *# *Microporus flabelliformis* clumped on broadleaf down rot-wood or litters. Unknown-function for food or medicine.
195. *Cryptoporus voluatus* clumped on pine stem, also on dying fir, spruce stem or dead standing trees. medicinal.
196. *Stereum faeiatum* on broadleaf deadwood, down wood and stump. Unknown-function for food or medicine.
197. *Stereum purpureum* on poplar, Salix, oak-like broadleaf stem or stump. Unknown-function for food or medicine.
198. *Stereum hirsutum* on poplar, Salix-like broadleaf living stem or dead woods-branches or stumps. Medicinal.
199. *Gyrophana lacrymans* usually on timber stems. Medicinal.
200. *Auricularia auricula* clustered on broadleaf trees or rot-woods or fir trees. Eatable, medicinal.
201. *# *Tremella foleacea* spring or autumn clumped in broadleaf forest rot-wood. Eatable.
202. *# *Phallus inpudicus* summer-autumn after rain on forest lands, clumped or separated. Medicinal.
203. # *Lycoperdon wrightii* summer-autumn clustered on forest land. Unknown-function for food or medicine.
204. *Lycoperdon pyriforme* summer, autumn clumped, or sometimes separated on rot-wood or at stem base, sometimes on lands. Medicinal.
205. *Lycoperdon perlatum* summer-autumn after rain in forest gap land. Medicinal.

**Ascomycota**

1. *# Peziza sylvestris forest land. Eatable.*
2. *Spathularia flavida summer-autumn in spruce, fir conifer forest land, clumped between mosses usually. Eatable, Ectomycorrhizal*
